# Hetiamacin E and F, New Amicoumacin Antibiotics from *Bacillus subtilis* PJS Using MS/MS-Based Molecular Networking

**DOI:** 10.3390/molecules25194446

**Published:** 2020-09-27

**Authors:** Ting Wang, Qinpei Lu, Chenghang Sun, Dmitrii Lukianov, Ilya Andreevich Osterman, Petr Vladimirovich Sergiev, Olga Anatolievna Dontsova, Xinxin Hu, Xuefu You, Shaowei Liu, Gang Wu

**Affiliations:** 1 Institute of Medicinal Biotechnology, Chinese Academy of Medical Sciences and Peking Union Medical College, Beijing 100050, China; tingwang0707@imb.pumc.edu.cn (T.W.); luqinpei@imb.pumc.edu.cn (Q.L.); sunchenghang@imb.pumc.edu.cn (C.S.); huxinxin1985@163.com (X.H.); xuefuyou@imb.pumc.edu.cn (X.Y.); 2 Beijing Key Laboratory of Antimicrobial Agents, Institute of Medicinal Biotechnology, Chinese Academy of Medical Sciences and Peking Union Medical College, Beijing 100050, China; 3 Center of Life Sciences, Skolkovo Institute of Science and Technology, Moscow 143025, Russia; dmitrii.lukianov@skoltech.ru (D.L.); i.osterman@skoltech.ru (I.A.O.); petya@genebee.msu.ru (P.V.S.); dontsova@genebee.msu.su (O.A.D.); 4 Department of Chemistry, Lomonosov Moscow State University, Moscow 119992, Russia; 5 Shemyakin-Ovchinnikov Institute of Bioorganic Chemistry, Russian Academy of Sciences, Moscow 119992, Russia

**Keywords:** *Bacillus subtilis* PJS, amicoumacins, antibacterial activity, methicillin-resistant *Staphylococcus aureus* (MRSA), inhibitors of protein biosynthesis

## Abstract

To combat escalating levels of antibiotic resistance, novel strategies are developed to address the everlasting demand for new antibiotics. This study aimed at investigating amicoumacin antibiotics from the desert-derived *Bacillus subtilis* PJS by using the modern MS/MS-based molecular networking approach. Two new amicoumacins, namely hetiamacin E (**1**) and hetiamacin F (**2**), were finally isolated. The planar structures were determined by analysis of extensive NMR spectroscopic and HR–ESI–MS data, and the absolute configurations were concluded by analysis of the CD spectrum. Hetiamacin E (**1**) showed strong antibacterial activities against methicillin-sensitive and resistant *Staphylococcus epidermidis* at 2–4 µg/mL, and methicillin-sensitive and resistant *Staphylococcus aureus* at 8–16 µg/mL. Hetiamacin F (**2**) exhibited moderate antibacterial activities against *Staphylococcus* sp. at 32 µg/mL. Both compounds were inhibitors of protein biosynthesis demonstrated by a double fluorescent protein reporter system.

## 1. Introduction

With increasing concerns over the continuous development of bacterial resistance to antibiotic drugs, there is an urgent need to develop efficient antibiotics. Natural products (NPs) derived from microorganisms are a major source of new chemical entities with potential applications for drug discovery, especially as anticancer and anti-infective agents [1]. In the last 30 years, the drug discovery pace has gradually slowed down, leading to a dramatic reduction in the number of new molecules identified. The phenomenon is in part due to the costs behind high rates of the rediscovery of known metabolites. Facing the dilemma, innovation strategies and techniques have been developed to push the process of NPs-based drug discovery research in recent decades [2,3].

Molecular networking (MN) is a rapidly developed cheminformatic approach, which is based on the principles that structurally related molecules will be fragmented by MS/MS at a similar pattern [4,5]. The molecular networking enables the visualization of large datasets and the grouping of fragmented ions into clusters, using an algorithm to compare the similarity of the fragmentation spectra. This concept has been integrated as a central component of the Global Natural Products Social (GNPS) molecular networking platform, where compound class identification is performed against a large, community-acquired reference library of spectra, and novel chemical entities related to known molecules can be efficiently affiliated with characteristic chemical families in clusters, thereby expediting the discovery and characterization process [6]. In the last few decades, molecular networking has appeared as a rapidly emerging and developing a method for NPs discovery, helping researchers to decipher the metabolomic “dark matter” of the molecular world. A growing number of reports have described the application of molecular networking for effective chemical dereplication and novel NPs discovery [7,8,9]. For example, one successful application was the isolation of new monoterpene indole alkaloids obtained from the bark of *Geissospermum leave*, guided by a molecular networking-based dereplication strategy using an in-house database of monoterpene indole alkaloids [10].

Amicoumacins are a small group of polyketide synthase-nonribosomal peptide synthetase (PKS-NRPS) derived natural products produced predominantly by *Bacillus* strains. These compounds possess a unique chemical scaffold with an isopentyl unit bonded to a 3,4-dihydro-8-hydroxy-isocoumarin as the common nucleus [11]. The structural variations are ascribed to the differences in an aliphatic, carbohydrate-like chain connected with the dihydroisocoumarin nucleus by an amido bond. To date, more than 40 amicoumacin analogs have been reported and they exhibit a wide range of biological activities, such as antibacterial [12,13,14,15], antifungal [16], anticancer [17,18,19], anti-inflammatory [20], antiulcer and herbicidal effects [21,22]. Many amicoumacins showed excellent inhibitory activities against Gram-positive bacteria, especially the methicillin- or oxacillin-resistant strains of *Staphylococcus aureus* (MRSA or ORSA). The antibacterial mechanism of some amicoumacins was deeply explored, for example, amicoumacin A interferes with translation by stabilizing the interaction of mRNA with the small ribosomal subunit in bacteria, thereby representing a new class of protein synthesis inhibitor [23]. The antifungal and anticancer mechanism in eukaryotes is similar to the antibacterial mechanism in bacteria but with certain differences in the elements of the binding sites, which inhibits translation by affecting translation elongation in yeast and mammalian systems [24]. Therefore, amicoumacins possess a valuable potential for using as antibacterial agents and it is of high value to discover and design more novel structures of amicoumacins.

**Figure 1:**
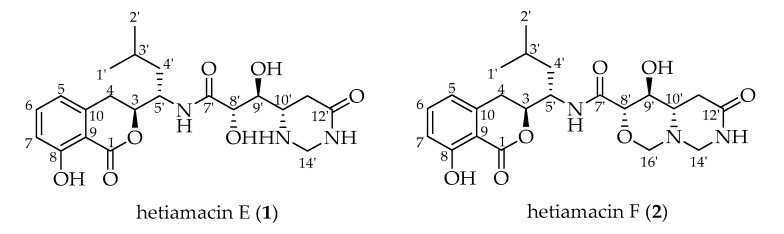
Chemical structures of hetiamacin E (**1**) and hetiamacin F (**2**).

*Bacillus subtilis* PJS is an endophytic bacterium isolated from a plant collected in the Hetian area in the Taklamakan desert, Xinjiang Uygur Autonomous Region, China [14]. In the previous study [12,14,25], a series of novel bioactive amicoumacins, hetiamacin A–D, have been obtained from this strain by means of traditional bioassay-guided separation. Recent advances in cheminformatics and analytical techniques spur us to deeply dig metabolites in the fermentation broth of strain PJS by using molecular networking-based dereplication strategy. This strategy applied in this study contributed to the discovery of two new amicoumacins from the same strain, which were designated as hetiamacin E (**1**) and hetiamacin F (**2**; Figure 1). The present research reported the MN-based dereplication of known amicoumacins from the fermentation broth of strain PJS, the MN-guided isolation, structural elucidation, evaluation of antibacterial activities, and mechanism determination of the two new amicoumacins.

## 2. Results

### 2.1. The Profile of Amicoumacins in Strain PJS Using MS/MS-Based Molecular Networking

The isolation of previously reported hetiamacin A−D and the development of a cheminformatic approach aroused our strong interest to further explore potential new amicoumacin entities from the fermentation broth of strain PJS. In order to quickly visualize and distinguish the secondary metabolites, molecular networking was employed to analyze amicoumacin congeners in the culture broth of strain PJS. The UPLC–MS/MS data were performed on the GNPS platform and displayed in Cytoscape (ver. 3.7.2). The entire network was presented in Figure S1. In the molecular network generated, 2278 individual MS/MS spectra were organized into 349 nodes, which formed 11 clusters of connected nodes, and these nodes possessed a wide range of precursor ions ranging from *m*/*z* 150 to 1000.

**Figure 2:**
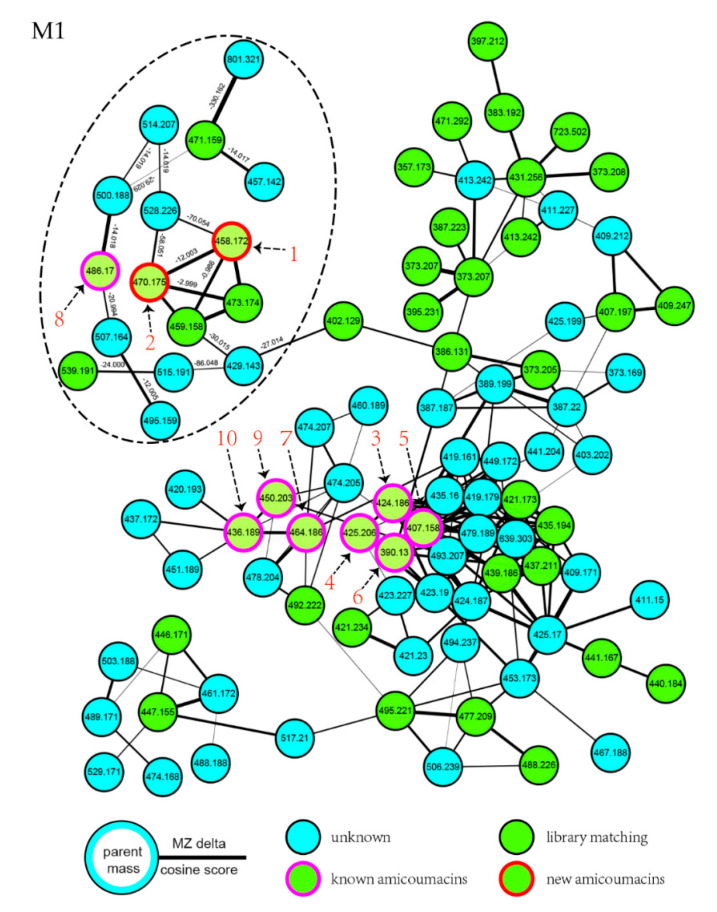
Molecular networking for the targeted fraction from the extract of *B. subtilis* PJS, contributing to the dereplication of known amicoumacins and directed discovery of new amicoumacin analogs (**1** and **2**). In the network, the experimental data of strain PJS extract nodes are represented by blue circles; nodes that are a consensus of experimental data and database information are shown as green circles; the nodes identified to be known amicoumacins are shown as green circles with magenta outer; and the nodes putative to be new amicoumacin analogs are shown as green circles with red outer.

**Figure 3:**
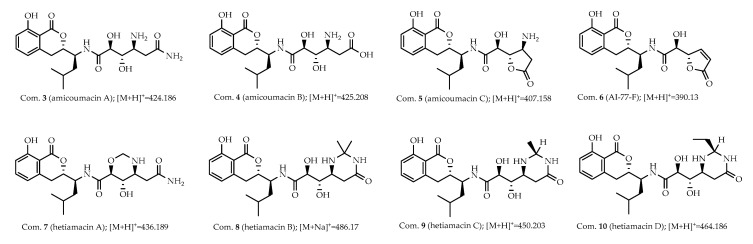
Known amicoumacins identified by using MS/MS-based molecular networking. Analog search and reference compound annotations with the previously identified amicoumacins (**3**−**10**) from strain PJS, and the corresponding spectral nodes were indicated with the numbers.

Further inspection of the molecular network aroused a great interest in the largest subnetwork (M1), which contained 95 spectral nodes with a strong spectral similarity score (cosine score > 0.6). Among these nodes, 30 nodes matched with the compounds in GNPS’ spectral libraries. As depicted in Figure 2, the neighboring nodes **3**−**10** were identified to be eight amicoumacin analogs, amicoumacin A (**3**), amicoumacin B (**4**), amicoumacin C (**5**), AI-77-F (**6**), hetiamacin A (**7**), hetiamacin B (**8**), hetiamacin C (**9**), and hetiamacin D (**10**; Figure 3), according to their molecular formulas predicted from the precursor ion *m*/*z* values (Figure S2), the characteristic of UV spectra (Figure S2) and MS/MS fragmentation pattern (Figure S3a,b; Table S1). Further analysis of the molecular network, we could find the compound **8** was remotely connected to other identified amicoumacins and formed a far branch (circled by the dotted oval). Thus, the nodes in the far branch aroused greater interest. For the nodes in the far branch, several compounds were potential of interest for isolation in consideration of their spectrum characteristics and putative novelty. Meanwhile, the LC−DAD−MS analysis of the fraction indicated that only two new putative amicoumacin analogs at *m*/*z* 458.172 [C_21_H_29_N_3_O_7_Na]^+^ and 470.175 [C_22_H_29_N_3_O_7_Na]^+^ could be isolated in amounts suitable for full structural elucidation ( Figure 1 and Figure S2).

### 2.2. Structure-Based Purification of Molecules Detected by Molecular Networking

With guidance by the above MS/MS-based molecular networking analysis, a large-scale fermentation of strain PJS was carried out in order to isolate the putative new analogs (**1** and **2**). The crude fraction (308 mg) obtained was firstly analyzed by UPLC–DAD–MS, and then applied for a step-by-step chromatographic isolation workflow. Under the structure-based purification, two new amicoumacin analogs **1** (1.8 mg) and **2** (2.5 mg) were obtained and their structural identifications are described below.

### 2.3. Structural Elucidation of Compounds

**Figure 4:**
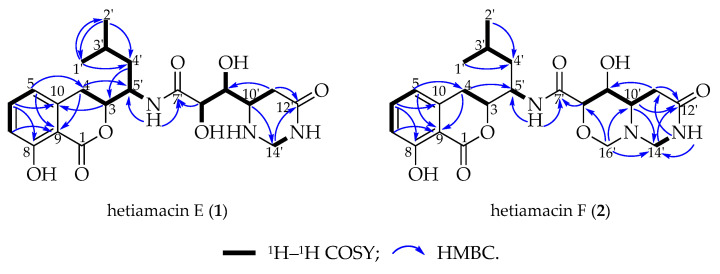
Major 2D NMR correlations of hetiamacin E (**1**) and hetiamacin F (**2**).

**Figure 5:**
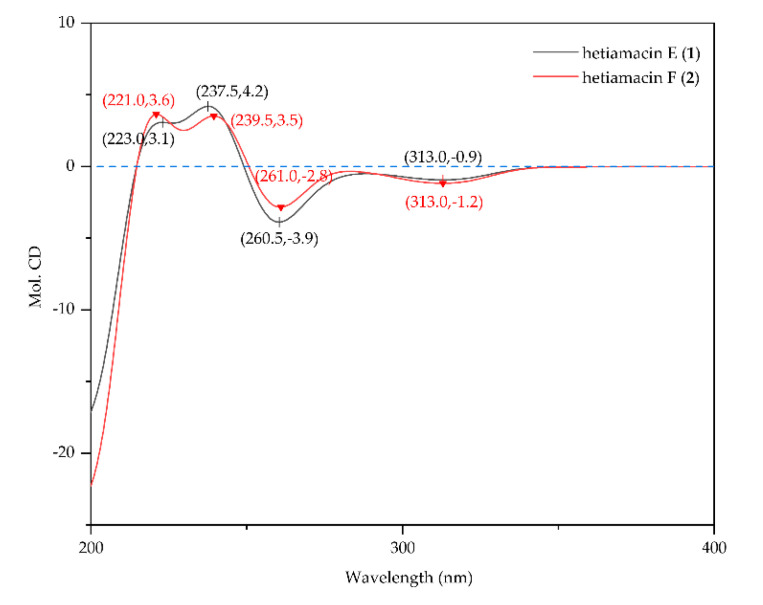
CD spectra of hetiamacin E (**1**) and hetiamacin F (**2**).

Compound **1** was isolated as a white amorphous solid, and its molecular formula was determined as C_21_H_29_N_3_O_7_ on the basis of the HR–ESI–MS (Figure S4) at *m/z* 458.1908 [M + Na]^+^ (calcd. for C_21_H_29_N_3_O_7_Na, 458.1903), accounting for nine degrees of unsaturation. Comparative analysis of ^13^C NMR in methanol-*d_4_* and DMSO-*d*_6_ (Figures S6 and S11) indicated that a methylene carbon signal (*δ*_C_ 40.8) was covered in DMSO-*d*_6_ solvents peaks. The relevant protons were assigned at their equivalent carbons after the analysis of ^1^H NMR, ^13^C NMR, and HSQC spectra (Figures S5–S7, S10–S11, Table 1). Analyzing the data of ^1^H–^1^H COSY spectrum (Figure S8), three spin systems were established. The first spin system was an aromatic ABC system for three aromatic proton signals at *δ*_H_ 7.49 (1H, t, H-6), 6.85 (1H, d, H–7), and 6.83 (1H, d, H–5). The remaining aromatic carbons were determined by analyzing relevant peaks from H–6 to C–10 (*δ*_C_ 140.7), from H–5 and H–7 to C–9 (*δ*_C_ 108.3), and from H–6 to C–8 (*δ*_C_ 160.8) in the HMBC spectrum (Figure 4 and Figure S9). The second spin system was a (CH_3_)_2_CHCH_2_CHCHCH_2_ (C−1′/C−2′ to C−4) structural fragment, which could be deduced by ^1^H–^1^H COSY correlations of H_3_−1′(H_3_−2′)/H−3′, H−3′/H_2_−4′, H_2_−4′/H−5′, H−5′/H−3, and H−3/H_2_−4. Then this coupling group was connected to C–10 based on the HMBC correlations from H–3 to C–10, from H–5 to C–4, and from H–4 to C–9. According to the chemical shift, C–3 (*δ*_C_ 81.1) should be a part of the lactone. Apart from these, the UV absorption at 197, 247, and 315 nm was identical to the characteristic UV spectra of amicoumacins [13]. Herein, the partial structure of an isocoumarin was determined, in which C–8 was substituted by a hydroxy group according to the singlet proton signal at *δ*_H_ 10.78 (1H, s, OH–8) in the downfield region of ^1^H-NMR. In the third spin system, the presence of the partial structure −CHCHCHCH_2_− (C−8′ to C−11′) was inferred by ^1^H–^1^H COSY correlations of H−8′/H−9′, H−9′/H−10′, and H−10′/H_2_−11′. In addition, the ^1^H–^1^H COSY relationship between H–8′ (*δ*_H_ 3.90) and 8′–OH (*δ*_H_ 5.57), together with the relationship between H–9′ (*δ*_H_ 3.68) and 9′–OH (*δ*_H_ 4.99), indicating the presence of an adjacent diol moiety. Through analysis of the HMBC data, C–11′ (*δ*_C_ 32.8) was linked to C–12′ (*δ*_C_ 169.4) based on the cross peak between H–11′ and C–12′. The C–14′ (*δ*_C_ 56.7) was bonded to two amino groups that were boned to C–10′ and C–12′, respectively, according to the COSY relationship between H–14′ and NH–13′, in addition to the HMBC correlations from H–10′ to C–14′, and from H–14′ to C–12′. Subsequently, C–8′ was related to C–7′ (*δ*_C_ 172.7) based on the cross peak between H–8′ and C–7′ in the HMBC spectrum. This unit was connected with the determined isocoumarin at C–5′ via an amide group according to the interactions from 6′–NH to C–7′ and C–5′ in the HMBC spectrum. Hence, the planar structure of compound **1** was established. The CD spectrum was tested to further determine the absolute configuration of compound **1**. In the CD spectrum (Figure 5 and Figure S12), compound **1** exhibited positive Cotton effects at 223.0 and 237.5 nm and negative Cotton effects at 260.5 and 313.0 nm. These effects were well in correspondence with the other previously reported amicoumacins (bacillcoumacins A–G) [15,26], therefore, the ultimately structure of compound **1** was established as illustrated in Figure 1.

Compound **2** was isolated as a white amorphous solid, and its molecular formula was determined as C_22_H_29_N_3_O_7_ on the basis of the HR–ESI–MS (*m*/*z* 470.1910 [M + Na]^+^, calcd. for C_22_H_29_N_3_O_7_Na, 470.1903) and NMR data (Figures S13–S21), demonstrating 10 degrees of unsaturation. The NMR spectral data (Table 1) of compound **2** is similar to compound **1** with an exception of the discrepancy caused by incremental methylene marked as C–16′ (*δ*_C_ 81.5) in the side chain of the amino acid. The HMBC correlations (Figure 4) from H–16′ to C–14′ (*δ*_C_ 57.6), C–10′ (*δ*_C_ 59.2) and C–8′ (*δ*_C_ 81.0) demonstrated that a ring was formed by the connection from C–16′ to 15′–N and 17′–O. Besides, the chemical shifts of C–8′ and C–9′ also compensated for this change. In the NOESY spectrum (Figure S21), the correlation between H–8′ and H–10′ confirmed that H–8′ and H–10′ were on the same side of the 1,3-oxazinane ring. By tracing the correlations from both H–8′ and H–14′ (*δ*_H_ 3.66) to H–16′ (*δ*_H_ 3.95), the stereogenic center C–8′ and C–10′ was assigned to S configuration. The CD spectrum (Figure 5 and Figure S22) of compound **2** was in good agreement with those for compound **1**, which demonstrated that the absolute configuration was consistent with compound **1**. Thus, the structure of compound **2** was established as depicted in Figure 1.

### 2.4. Antibacterial Activity

Both compounds were evaluated for antibacterial activities against a series of Gram-staining-positive bacteria and Gram-staining-negative bacteria by the agar dilution method. As shown in Table 2, hetiamacin E (**1**) exhibited strong activities against strains of *Staphylococcus* spp. The inhibitory activities against methicillin-sensitive and resistant *Staphylococcus epidermidis* (*S. epidermidis*) with MIC values of 2–4 µg/mL were more active than that of *Staphylococcus aureus* (*S. aureus*) with MIC values of 8–16 µg/mL. As for hetiamacin F (**2**), only weak inhibitory activities (32 µg/mL) were detected against *S. epidermidis* ATCC 12228, *S. epidermidis* 16-5, *Pseudomonas aeruginosa* PAO1, and *Acinetobacter baumannii* ATCC 19606. Regarding other tested bacteria shown in Table S2, such as *Enterococcus faecalis* ATCC 29212 (VSE), *Klebsiella pneumonia* ATCC 700603, and *Enterobacter cloacae* ATCC 43560, etc., the bacteria growth was not affected by the maximum concentrations of the tested antibacterial agents used in this work.

### 2.5. Mechanism of Action Determination

**Figure 6:**
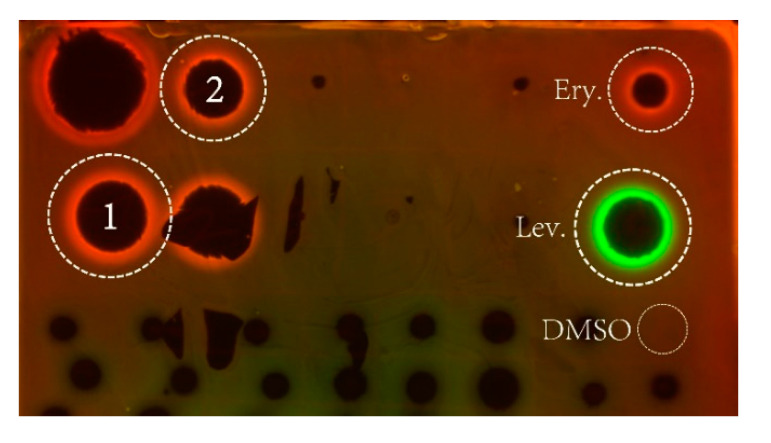
Agar plate coated with a layer of reporter strain JW5503 (Δ*tolC*) of *E. coli* that was transformed by plasmid pDualrep2. Spots of erythromycin (Ery), levofloxacin (Lev), DMSO, and Compounds 1 and 2) were placed on the surface of the agar plate. The plate was scanned in the Cy3 and Cy5 channels to determine RFP andKatushka2S fluorescence, respectively, and showed a combined image where RFP and Katushka2S fluorescence were shown in green and red hues, respectively. Fluorescence of RFP protein-induced DNA damaged SOS response; while, Katushka2S indicates translation inhibition.

The mechanism of antibacterial activity of hetiamacin E (**1**) and hetiamacin F (**2**) were determined by using the pDualrep2 reported system [27]. As shown in Figure 6, both compounds induced Katushka2S expression as erythromycin did, which demonstrated these two compounds were all protein biosynthesis inhibitors that could cause ribosome stalling during mRNA translation.

## 3. Discussion

*Bacillus* species have been well known to produce structurally diverse classes of secondary metabolites, such as lipopeptides, polypeptides, macrolactones, fatty acids, polyketides, lipoamides, and isocoumarins [28,29]. Among these metabolites, amicoumacin-based isocoumarins have evoked great interest in medicinal research because of their wide spectrum of biological activities. Up to now, about 30 strains of the genus *Bacillus* were described as amicoumacin producers, among them, *B. subtilis* strains were mostly reported [30]. Notably, *B. subtilis* strains isolated from different biotopes or geographic origins could produce versatile structures of amicoumacin compounds [31]. For example, thermotolerant bacterium *B. subtilis* KFSB5 isolated from the compost soil of Kinwat teak forest in India could produce amicoumacin A—like isocoumarin antibiotics; strain *B. subtilis* 3 isolated from animal fodder in Ukraine could produce amicoumacin A, B, and C [32]; strain *B. subtilis* TP-B0611 isolated from sardine intestine collected in Toyama Bay in Japan could produce bacilosarcins A and B [21]; strain *B. subtilis* B1779 isolated from sediment of the Red Sea could produce bacisarcin C and lipoamicoumacins A–D [15]. Unlike previously reported *B. subtilis*, strain PJS was the first amicoumacin producer isolated from the desert environment. It was reported that there was a great difference between the biosynthetic capabilities of different *B. subtilis* strains from various sources [33]. This intraspecific diversity of secondary metabolite production is most likely related to horizontal gene transfer enabled by the natural competence of *B. subtilis* under the stress of survival environments [19,29,33]. The discovery of specific compounds hetiamacin E (**1**) and F (**2**), along with the previously reported hetiamacin A–D further verified the genetic heterogeneity and metabolites diversity of *B. subtilis* colonized in different habitats. Thus, the biosynthetic potential of strain PJS is of great value for further studies.

The aim of this research was to deeply investigate the novel metabolites in fermentation extracts of *B. subtilis* PJS by using a recently developed cheminformatic approach, molecular networking [4]. As a visualization tool designed to address structural elucidation of different compounds, molecular networking is compatible with early identification of new molecules that are related to known substances and provides opportunities to identify new molecular families from complex mixtures [34,35,36]. The application of molecular networking in our latest studies have successfully contributed to the discovery of some new chemical entities, such as the discovery of new streptogramin-type antibiotics, acetyl-griseoviridin, and desulphurizing griseoviridin in the fermentation broth of *Streptomyces* sp. 8P21H-1, and the speculation about the existence of five putative new quinoxaline-type antibiotics in the fermentation broth of *Streptomyces* sp. B475 [37,38]. Comparison of traditional untargeted or bioassay-guided approaches, molecular networking is fast, requiring minimal time and sample material. In the previous research conducted on the metabolites of stain PJS, owing to the limit of the bioassay-guided approach, the new components that are low in amounts and/or high in the detection limit of activity are easy to be overlooked. The use of molecular networking resulted in quick recognition of all amicoumacin derivatives in the fermentation broth of strain PJS as one integrated subnetwork. The analysis of the network combined with spectrum characteristics triggered the structural elucidation of closely related nodes, which led to the identification of the known amicoumacins and the isolation of two new amicoumacins (**1** and **2**).

Both hetiamacin E (**1**) and hetiamacin F (**2**) exhibited selectively antibacterial activities against some Gram-positive bacteria, especially against MRSA and MRSE. Preliminary research on the mechanism of antibacterial activity with a double fluorescent reporter system demonstrated that both compounds exerted their antibacterial effects by inhibiting protein translation. This finding was corresponding to the antibacterial mechanism of amicoumacin A, which is the most and the earliest studied among amicoumacin compounds. The mechanism action of amicoumacin A is targeting the synthesis of protein by binding a conserved site between the E-site mRNA codon and 16S rRNA, hampering the movement of mRNA relative to the small ribosomal subunit [23]. The long hydrophilic 8′,9′-dihydroxy-10′-aminohexandiamide tail of amicoumacin A formed multiple hydrogenbonds with the conserved 16S rRNA bases U788, A794, and C795 in the loop of the helix 24; the terminal amide moiety forms magnesium-mediated contacts with the phosphate groups of nucleotides G1505 and U1506 [23]. Therefore, it was concluded that the hydroxyl or amino group at C−8′, C−9′, C–10′ and the amide group (C–12′) should be the key pharmacophores for the antibacterial activity of amicoumacins. The conclusion was further supported by a comparison of the structure–activity relationship between amicoumacin A and B [20,22], between bacilosarcins A, B, and C, etc. [15,21]. In the present study, both hetiamacin E (**1**) and hetiamacin F (**2**) possessed the same skeleton as amicoumacin A but with different degrees of cyclization action between 8′-hydroxy group and 12′-amide group at the side chain, therefore, it was inferred that deactivation of these free pharmacophores leads to their lower antibacterial activities than amicoumacin A. In addition, the discrepancy of antibacterial activities between hetiamacin E (**1**) and hetiamacin F (**2**) should be also attributed to the formation of the oxazinane ring.

## 4. Materials and Methods

### 4.1. General Procedures

The Diaion HP-20 was purchased from Mitsubishi Chemical Holdings Corp., Tokyo, Japan. The syringe filter units (0.22 µm polytetrafluoroethylene membranes) were purchased from Tianjin Jinteng Experiment Equipment Co. Ltd., Tianjin, China). Low-resolution electrospray ionization (ESI) mass spectra were measured on UPLC–DAD–MS system (LC-20AD, SPD-M20, and LCMS-2020, Shimadzu Corp., Tokyo, Japan) equipped with an Eclipse XDB-C18 column (4.6 × 150 mm, i.d. 5 µm; Agilent, Santa Clara, CA, USA). High-resolution ESI–MS data were obtained using UPLC-HRESIMS/MS instrument (Waters XevoG2-XS QTof, Waters Co., Wilmslow, UK) equipped with ACQUITY UPLC BEH C18 column (2.1 × 100 mm, 1.7 µm). Reverse-phase medium pressure flash liquid chromatography (RP-MPLC) was conducted on an Isolera^TM^ One system (Biotage, Uppsala, Sweden) using a LiChroprep RP-C18 column (50 × 1.0 cm, 40–63 µm, Merck, Darmstadt, Germany). The semipreparative HPLC separations were performed on a prominence system (LC-20AT, Shimadzu, Tokyo, Japan) instrument equipped with a binary pump and a UV-visible diode array detector (190−800 nm) using the ZORBAX SB-C18 column (9.4 × 250 mm, i.d. 5 µm; Agilent). One-dimensional (1D) and 2D NMR spectra were recorded on Bruker AM-500 (500 MHz) and AM-600 (600 MHz). Samples were dissolved in 0.2 mL DMSO-*d*_6_ as solvent and transferred to a 3 mm NMR tube. Chemical shifts were recorded in ppm (*δ* unit) and referenced to residual solvents signals.

### 4.2. Small-Scale Fermentation and Preliminary UPLC-UV Analysis

The preparation of the agar slant and cultural broth of strain PJS were the same as described previously [14]. The strain was inoculated into three 500 mL Erlenmeyer flasks containing 100 mL of modified Gause’s No.1 cultural broth (soluble starch 20.0 g, NaCl 50.0 g, K_2_HPO_4_ 0.5 g, KNO_3_ 1.0 g, MgSO_4_ 1.0 g, FeSO_4_ 0.02 g, glucose 1.0 g, peptone 0.5 g, tryptone 0.3 g, and *N*-acyl-asparagine 0.5 g in 1.0 L distilled water, pH 8.0), and cultured on a rotary shaker at 180 rpm and 28 °C. After 3 days of fermentation, the cultural broth (300 mL) was centrifuged at 4500 rpm to separate the mycelia and supernatant. Subsequently, the supernatant was absorbed on a column containing 30 mL of Diaion HP-20 and was eluted progressively with 0%, 20%, 40%, 60%, and 80% acetone to give different fractions. Each fraction was applied for preliminary UPLC-UV analysis. The fractions rich in components of amicoumacins with characteristic UV spectral features (maximum absorption wavelength at 246 nm and 314 nm) were combined and concentrated in vacuo to give a brown-gray solid (18 mg). The obtained brown-gray solid was dissolved in methanol (500 µL) and filtered through a syringe filter unit to get a filter liquor (500 µL) for further mass spectrometry analysis.

### 4.3. Mass Spectrometry Analysis

To deeply explore novel amicoumacin analogs, the filter liquor (500 µL) was reanalyzed on a Waters Xevo G2-XS QTof mass spectrometer system to generate a MS/MS-based molecular network. A gradient of acetonitrile-water (A: H_2_O, B: acetonitrile) was employed with a flow rate of 300 µL/min on an ACQUITY UPLC BEH C18 column. The gradient started from 30% B for 1 min, followed by a linear gradient to reach 50% B for 10 min, another linear gradient to 90% B for 3 min, finally held for 2 min at 90% B. ESI source operated in positive ion mode and conditions were set as below: the source temperature at 100 °C, the desolvation temperature at 250 °C, capillary voltage at 2.0 kV, sample cone voltage at 40.0 V, cone gas flow rate of 45 L/h, and desolvation gas flow rate of 600 L/h. Data-dependent acquisition (DDA) mode was selected for MS acquisition, the full MS survey scan range was performed from 100 to 1500 Da for 0.1 s. MS^2^ fragmentation scan was performed over the range from 100 to 1500 Da in the same scan time. The 10 most intense ions were further scanned for MS/MS fragmentation spectra. The gradient of collision energy was set as 6 to 9 V for LM CE (low-mass collision energy) and 60 to 80 V for HM CE (high-mass collision energy). Low mass (LM) was set as 100 Da and high mass (HM) was set as 1500 Da.

### 4.4. Molecular Networking

The MS data of the targeted fraction was converted from the raw format to the mzXML format using the Proteo Wizard tool [39]. Then, the converted data file was uploaded by the suggested software of WinSCP (http://winscp.net/eng/download.php) to the GNPS platform (https://gnps.ucsd.edu) developed by Mingxun Wang and coworkers [40]. The resulting analysis and parameters for the network can be accessed via the link https://gnps.ucsd.edu/ProteoSAFe/status.jsp?task = 6a4c9cd3db7746d9a256138b51e50dbf. Raw MS/MS data were deposited in the MassIVE Public GNPS data sets (https://massive.ucsd.edu) with accession no. MSV000086181. The following settings were used for generation of the network: minimum pairs cos 0.6; precursor ion mass tolerance, 0.02 Da; MS/MS fragment ion tolerance, 0.02 Da; network topK, 10; minimum matched peaks, 2; minimum cluster size, 10. The molecular networking data were analyzed and visualized using Cytoscape (ver. 3.7.2) [41]. A detailed analysis of the molecular network led to the chemical targeting of hetiamacin E (**1**) and hetiamacin F (**2**) based on the network cluster with their associated molecular mass (*m/z* 458 and 470).

### 4.5. Large-Scale Fermentation, Extraction, and Targeted Isolation

On the basis of the guidance of molecular networking, large-scale fermentation of *B. subtilis* PJS was further conducted. The strain PJS was seeded in 500 mL Erlenmeyer flasks that contained 100 mL of modified Gause’s No.1 broth and cultured on a rotary shaker at 180 rpm and 28 °C for 2 days. Subsequently, the seed culture was transferred into 5 L flasks containing 1 L of modified Gause’s No.1 broth mentioned above and incubated for 4 days in the same condition. The whole fermentation broth (20 L) was centrifuged and the supernatant was performed on a column filled with Diaion HP-20 (2 L). The column was eluted with different gradient acetone-water (0:100, 20:80, 40:60, 60:40, and 80:20, *v*/*v*). Each fraction was applied to UPLC–DAD–MS analysis to monitor or test the appearance of the putative new metabolites at *m*/*z* 458 and 470. The fractions containing putative new metabolites (**1** and **2**) were merged and concentrated in vacuo to afford crude extract (308 mg). After dissolved in methanol (10 mL), the crude extract was separated by reversed-phase medium pressure flash liquid chromatography (RP-MPLC) and eluted with a gradient of methanol/water (30–100% methanol) over 10 min at a flow rate of 8 mL/min. Then, the fractions were reanalyzed by the UPLC–DAD–MS system to gather the fractions that contained putative new metabolites, which gave a brown material (50 mg). After dissolved in 5 mL methanol, the sample was subjected to semipreparative HPLC and eluted with methanol/water (a flow rate of 2.0 mL/min, UV detection at 246 and 314 nm, 50% MeOH in 50 min) to yield compound **1** (1.8 mg, 22.2 min), and compound **2** (2.5 mg, 26.6 min) respectively.

### 4.6. Antibacterial Assay

The antibacterial activities of compounds **1** and **2** were determined by agar dilution methods according to CLSI guidelines [42]. Bacteria strains used in this study were either purchased from American Type Culture Collection (ATCC) or obtained from the clinic and deposited in the Institute of Medicinal Biotechnology, Chinese Academy of Medical Sciences and Peking Union Medical College. Levofloxacin was used as positive control, and DMSO was used as negative control. Compounds **1** and **2** were dissolved in DMSO and two-fold serially diluted in Mueller–Hinton agar. The final concentrations of compounds ranged from 0.125 to 64 µg/mL for compound **1**, and from 0.125 to 32 µg/mL for compound **2**. Bacterial strains tested were incubated in Mueller–Hinton broth for 12 h and then diluted to 0.5 McFarland standard (approximately 10^8^ CFU/mL). After 100 folds dilution, the tested bacteria (approximately 10^6^ CFU/mL) were inoculated on Mueller–Hinton agar plates by the MIT-P multipoint inoculator (Sakuma, Tokyo, Japan). After incubation at 37 °C for 18 h, the MICs were recorded.

### 4.7. Mechanism of Action Determination

The antibacterial mechanisms of compounds **1** and **2** were tested by a double fluorescent protein reporter system with JW5503-pDualrep2 as the reporter strain [27]. Specifically, compound **1** (0.5 mg) and **2** (0.5 mg) were dissolved in 0.1 mL DMSO, and then 2 µL of each compound was spotted on an agar plate containing a lawn of the reporter strain. After overnight incubation at 37 °C, the plate was scanned by ChemiDoc Imaging System (Bio-Rad, Hercules, CA, USA) with two channels, “Cy3-blot” (553/574 nm, green pseudocolor) for RFP fluorescence and “Cy5-blot” (588/633 nm, red pseudocolor) for Katushka2S fluorescence. Induction of expression of Katushka2S is triggered by translation inhibitors, while RFP is upregulated by induction of DNA damaged SOS response. Levofloxacin (Lev.) and erythromycin (Ery.) were used as positive controls for DNA biosynthesis and ribosome inhibitors, respectively, and DMSO was used as negative control.

## 5. Conclusions

This research provided a typical example of the MS/MS-based molecular networking method aided in natural product discovery efforts. The use of molecular networking to supervise substructure annotation indicated the presence of new amicoumacin antibiotics (**1** and **2**) in the fraction of strain PJS, and the structure-guided isolation procedure contributed to obtaining these two compounds. Compound **1** exhibited strong activities against methicillin-sensitive and resistant *S. epidermidis* with MIC value of 2–4 µg/mL, compound **2** exhibited moderate activity against *Staphylococcus* spp. at 32 µg/mL. Both compounds were demonstrated to be protein biosynthesis inhibitors that could cause ribosome stalling during mRNA translation. The application of molecular networking enables a clear view of the structural breadth and provides rapid identification of and deep investigation of the novel analogs, and would drastically increase the chances of new antibiotics discovery and development.

## Figures and Tables

**Table 1:** NMR data of hetiamacin E (**1**) and hetiamacin F (**2**) in DMSO-*d*_6._

No.	Hetiamacin E (1) ^a^			Hetiamacin F (2) ^b^		
	*δ*_C_	*δ*_H_, Mult (*J* in Hz)	HMBC	*δ*_C_	*δ*_H_, Mult (*J* in Hz)	HMBC
1	169.1, C		7	169.1, C		
3	81.1, CH	4.70, d (12.5)	4,4′	81.2, CH	4.69, dt (12.8, 2.7)	4
4	29.1, CH_2_	3.08–3.02, m; 2.90–2.82, m	5	29.2, CH_2_	3.00, dd (16.3, 13.0); 2.82, dd (16.7, 2.8)	5
5	118.5, CH	6.83, d (7.3)	7,4	118.6, CH	6.82, d (7.5)	7,4
6	136.3, CH	7.49, t (7.9)	5	136.3, CH	7.51–7.47, m	
7	115.2, CH	6.85, d (8.4)	5	115.3, CH	6.85, d (8.4)	5
8	160.8, C		6,7	160.9, C		6
8–OH		10.78, s			10.82, s	
9	108.3, C		6,5,7,4	108.3, C		7,5,4
10	140.7, C		6,5,3,4	140.7, C		6,4
1′	21.5, CH_3_	0.85, d (6.2)	4′,2′	21.5, CH_3_	0.86, d (6.5)	2′,4′
2′	23.3, CH_3_	0.89, d (6.4)	1′	23.3, CH_3_	0.91, d (6.6)	4′,1′
3′	23.9, CH	1.67–1.59, m	4′,1′,2′	24.1, CH	1.67–1.58, m	
4′	40.0, CH_2_	1.73–1.67, m; 1.36–1.28, m	3, 1′, 2′	40.6, CH_2_	1.75–1.67, m; 1.38–1.31, m	1′,2′
5′	47.9, CH	4.24–4.14, m	6′–NH,4,4′	48.2, CH	4.24–4.18, m	4,4′
6′–NH		7.64, d (9.5)			8.12, d (9.2)	
7′	172.7, C		6′–NH,8′	168.9, C		6′-NH,8′
8′	72.3, CH	3.90, d (6.6)	8′–OH	81.0, CH	3.72, d (9.2)	16′
8′–OH		5.57, s				
9′	73.7, CH	3.68, d (5.7)	8′,10′,11′	65.9, CH	3.50–3.44, m	8′,10′,11′
9′–OH		4.99, d (5.6)			5.29, d (6.2)	
10′	52.6, CH	3.02–2.95, m	14′,8′,9′,11′	59.2, CH	2.63–2.57, m	16′,14′,11′
11′	32.8, CH_2_	2.13–2.07, m	9′,13′–NH	32.9, CH_2_	2.43, dd (17.7, 6.2); 2.21, dd (17.7, 6.2)	13′-NH
12′	169.4, C		11′,13′−NH,14′	167.7, C		14′,10′,11′
13′–NH		7.61, s			7.90, s	
14′	56.7, CH_2_	4.03–3.95, m	10′,13′–NH	57.6, CH_2_	4.17, dd (9.0, 2.6); 3.66, dd (9.0, 2.1)	16′, 10′, 13′
15′–NH		not detected				
16′				81.5, CH_2_	4.51, d (9.1); 3.95, d (9.2)	

^a^ 500 MHz for ^1^H-NMR, 125 MHz for ^13^C-NMR. ^b^ 600 MHz for ^1^H-NMR, 150 MHz for ^13^C-NMR.

**Table 2:** Minimum inhibitory concentrations (MICs) of hetiamacin E (**1**) and hetiamacin F (**2**).

Test Organisms	MICs (µg/mL)		
	Hetiamacin E (1)	Hetiamacin F (2)	Levofloxacin
*S. epidermidis* ATCC 12228 (MSSE)	2	32	0.25
*S. epidermidis* 16-4 (MSSE)	4	32	0.25
*S. epidermidis* 16-5 (MRSE)	4	32	8
*S. aureus* ATCC 29213 (MSSA)	8	>32	0.25
*S. aureus* ATCC 33591 (MRSA)	16	>32	0.25
*Enterococcus faecium* ATCC 700221 (VRE)	64	>32	32
*Escherichia coli* ATCC 25922	64	>32	≤0.03
*Pseudomonas aeruginosa* PAO1	64	32	4
*Acinetobacter baumannii* ATCC 19606	64	32	0.25
*Shigella flexneri* ATCC 12022	64	>32	≤0.03

Note: MSSE, methicillin-susceptible *Staphylococcus epidermidis*; MRSE, methicillin-resistant *Staphylococcus epidermidis*; MSSA, methicillin-susceptible *Staphylococcus aureus*; MRSA, methicillin-resistant *Staphylococcus aureus*; VRE, vancomycin-resistant *Enterococcus*.

## Supplementary Materials

The following are available online. Figure S1: The entire network generated by the MS/MS data from extract of strain PJS, Figure S2: UPLC-UV-MS chromatogram of crude extract from strain PJS, Figure S3a: The MS/MS spectrum and possible fragmentation patterns of compounds **1**–**5**, Figure S3b: The MS/MS spectrum and possible fragmentation patterns of compounds **6**–**10**, Figure S4: The HR-ESI-MS spectrum of compound **1**, Figure S5: The ^1^H NMR spectrum of compound **1** in DMSO-*d*_6_ (500 MHz), Figure S6: The ^13^C NMR spectrum of compound **1** in DMSO-*d*_6_ (125 MHz), Figure S7: The HSQC spectrum of compound **1** in DMSO-*d*_6_, Figure S8: The COSY spectrum of compound **1** in DMSO-*d*_6_, Figure S9: The HMBC spectrum of compound **1** in DMSO-*d*_6_, Figure S10: The ^1^H NMR spectrum of compound **1** in CD_3_OD (500 MHz), Figure S11: The ^13^C NMR spectrum of compound **1** in CD_3_OD (125 MHz), Figure S12: The CD spectrum of compound **1**, Figure S13: The HR-ESI-MS spectrum of compound **2**, Figure S14: The ^1^H NMR spectrum of compound **2** in DMSO (600 MHz), Figure S15: The ^13^C NMR spectrum of compound **2** in DMSO-*d*_6_ (150 MHz), Figure S16: The DEPT 90 spectrum of compound **2** in DMSO-*d*_6_ (150 MHz), Figure S17: The DEPT spectrum 135 of compound **2** in DMSO-*d*_6_ (150 MHz), Figure S18: The HSQC spectrum of compound **2** in DMSO-*d*_6_, Figure S19: The COSY spectrum of compound **2** in DMSO-*d*_6_, Figure S20: The HMBC spectrum of compound **2** in DMSO-*d*_6_, Figure S21: The NOESY spectrum of compound **2** in DMSO-*d*_6_, Figure S22: The CD spectrum of compound **2**, Table S1: The MS/MS fragment ions of two new antibiotics (**1**–**2**) and compounds **3**–**10**, Table S2: Minimum inhibitory concentrations (MICs) of hetiamacin E (**1**) and hetiamacin F (**2**).
