# Supplementary material for: Hetiamacin E and F, New Amicoumacin Antibiotics from Bacillus subtilis PJS Using MS/MS-Based Molecular Networking
Source: Molecules. 2020 Sep 27;25(19):4446. doi: 10.3390/molecules25194446 (PMC7583885; doi:10.3390/molecules25194446)
Supplement: Supplementary file 1 [file molecules-25-04446-s001.docx]

Hetiamacin E and F, New Amicoumacin Antibiotics from *Bacillus subtilis* PJS Using MS/MS-Based Molecular Networking

Ting Wang ^1,2^, Qinpei Lu ^1,2^, Chenghang Sun ^1,2^, Dmitrii Lukianov ^3^, Ilya Andreevich Osterman ^3,4^, Petr Vladimirovich Sergiev ^3,4^, Olga Anatolievna Dontsova ^3,4,5^, Xinxin Hu ^1,2^, Xuefu You ^1,2^, Shaowei Liu ^1,2,^* and Gang Wu ^1,2,^*

^1^ Institute of Medicinal Biotechnology, Chinese Academy of Medical Sciences and Peking Union Medical College, Beijing 100050, China; tingwang0707@imb.pumc.edu.cn (T.W.); luqinpei@imb.pumc.edu.cn (Q.L.); sunchenghang@imb.pumc.edu.cn (C.S.); huxinxin1985@163.com (X.H.); xuefuyou@imb.pumc.edu.cn (X.Y.)

^2^ Beijing Key Laboratory of Antimicrobial Agents, Institute of Medicinal Biotechnology, Chinese Academy of Medical Sciences and Peking Union Medical College, Beijing 100050, China

^3^ Center of Life Sciences, Skolkovo Institute of Science and Technology, University, Moscow 143025, Russia; dmitrii.lukianov@skoltech.ru (D.A.L.); i.osterman@skoltech.ru (I.A.O.); petya@genebee.msu.ru (P.V.S.); dontsova@genebee.msu.su (O.A.D.)

^4^ Department of Chemistry, Lomonosov Moscow State University, Moscow 119992, Russia

^5^ Shemyakin-Ovchinnikov Institute of Bioorganic Chemistry, Russian Academy of Sciences, Moscow 119992, Russia

*****Correspondence: liushaowei@imb.pumc.edu.cn (S.L.); wugang@imb.pumc.edu.cn (G.W.); Tel.: +86–10–6316–5272 (S.L.); Tel.: +86–10–6316–5278 (G.W.)

**List of supplementary materials**

**Supplementary figures: page S3-S17.**

| **No.** | **Contents** | **Page** |
| --- | --- | --- |
| **Figure S1** | The entire network generated by the MS/MS data from extract of strain PJS. | **S3** |
| **Figure S2** | UPLC-UV-MS chromatogram of sample from strain PJS. | **S4** |
| **Figure S3a** | The MS/MS spectrum and possible fragmentation patterns of compounds **1**-**5**. | **S5** |
| **Figure S3b** | The MS/MS spectrum and possible fragmentation patterns of compounds **6**-**10**. | **S6** |
| **Figure S4** | The HR-ESI-MS spectrum of compound **1**. | **S7** |
| **Figure S5** | The ^1^H NMR spectrum of compound **1** in DMSO-*d_6_* (500 MHz). | **S8** |
| **Figure S6** | The ^13^C NMR spectrum of compound **1** in DMSO-*d_6_* (125 MHz). | **S8** |
| **Figure S7** | The HSQC spectrum of compound **1** in DMSO-*d_6_*. | **S9** |
| **Figure S8** | The COSY spectrum of compound **1** in DMSO-*d_6_*. | **S9** |
| **Figure S9** | The HMBC spectrum of compound **1** in DMSO-*d_6_*. | **S10** |
| **Figure S10** | The ^1^H NMR spectrum of compound **1** in CD_3_OD (500 MHz). | **S10** |
| **Figure S11** | The ^13^C NMR spectrum of compound **1** in CD_3_OD (125MHz). | **S11** |
| **Figure S12** | The CD spectrum of compound **1**. | **S11** |
| **Figure S13** | The HR-ESI-MS spectrum of compound **2.** | **S12** |
| **Figure S14** | The ^1^H NMR spectrum of compound **2** in DMSO-*d_6_* (600 MHz). | **S13** |
| **Figure S15** | The ^13^C NMR spectrum of compound **2** in DMSO-*d_6_* (150 MHz). | **S13** |
| **Figure S16**  **Figure S17** | The DEPT 90 spectrum of compound **2** in DMSO-*d_6_* (150 MHz).  The DEPT 135 spectrum of compound **2** in DMSO-*d_6_* (150 MHz). | **S14**  **S14** |
| **Figure S18** | The HSQC spectrum of compound **2** in DMSO-*d_6_*. | **S15** |
| **Figure S19** | The COSY spectrum of compound **2** in DMSO-*d_6_*. | **S15** |
| **Figure S20** | The HMBC spectrum of compound **2** in DMSO-*d_6_*. | **S16** |
| **Figure S21** | The NOESY spectrum of compound **2** in DMSO-*d_6_*. | **S16** |
| **Figure S22** | The CD spectrum of compound **2**. | **S17** |

**Supplementary tables: page S18-S19.**

| **No.** | **Contents** | **Page** |
| --- | --- | --- |
| **Table S1** | The MS/MS fragment ions of two new antibiotics (**1**-**2**) and compounds **3**-**10**. | **S18** |
| **Table S2** | Minimum inhibitory concentrations (MICs) of hetiamacin E (**1**) and hetiamacin F (**2**) | **S19** |


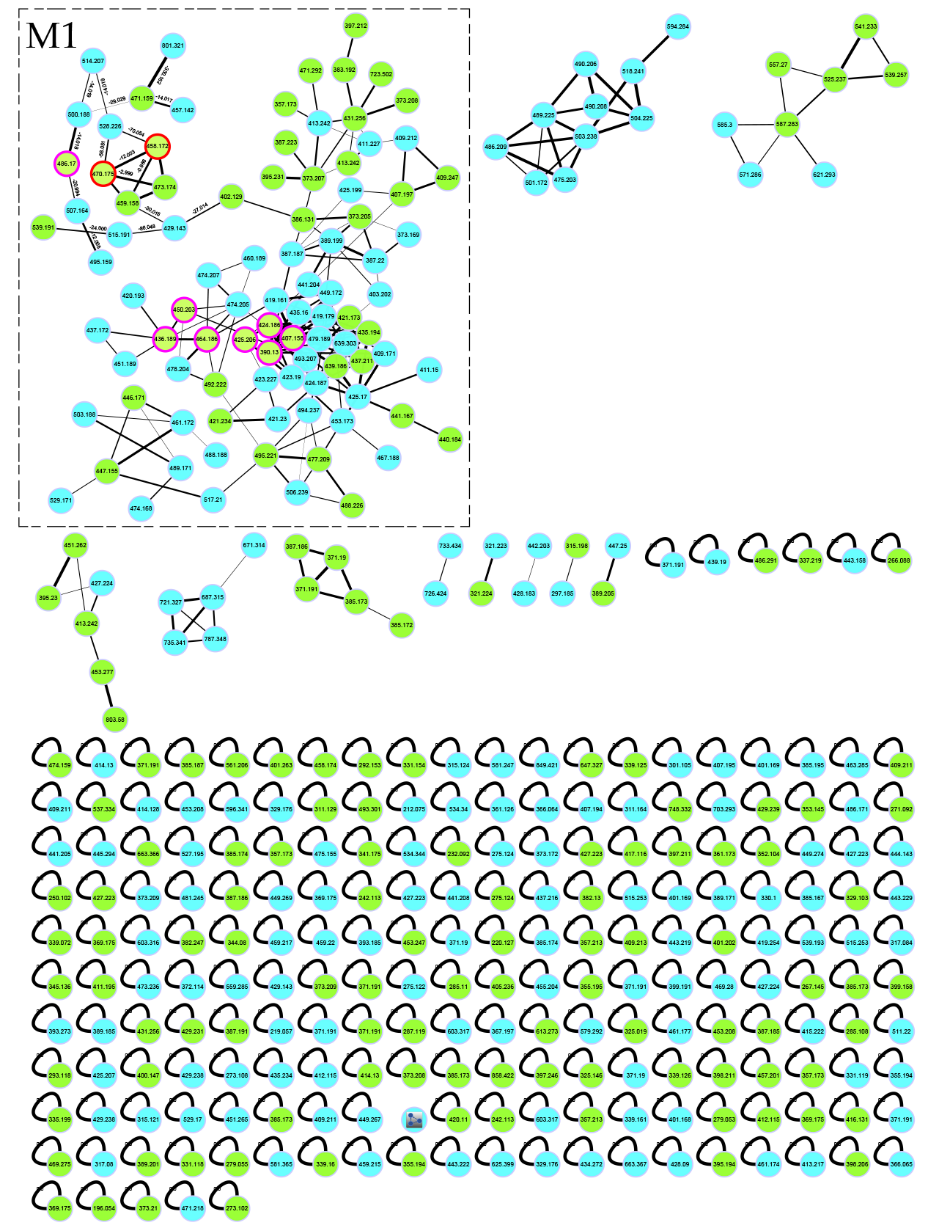


**Figure S1**. The entire network generated by the MS/MS data from extract of strain PJS. Cluster M1 was identified to be related with amicoumacins.


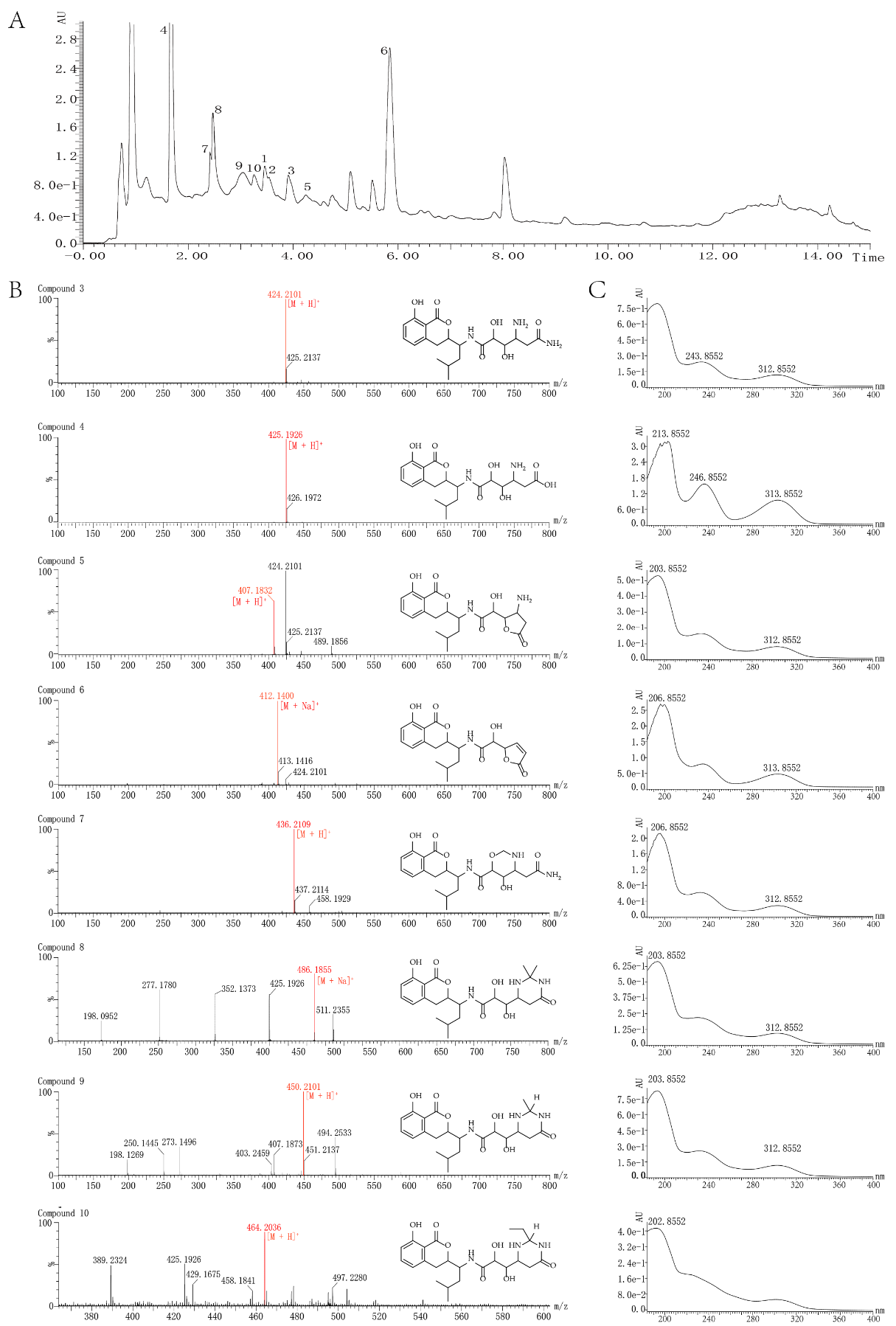


**Figure S2**. UPLC-UV-MS chromatogram of sample from strain PJS. (A) UPLC profile of the crude extract from strain PJS, eight identified amicoumacins and two new amicoumacin analogs were labelled in the UPLC spectrum. (B) The mass spectra of eight amicoumacins identified by molecular networking. (C) UV spectra of eight amicoumacins identified by molecular networking.


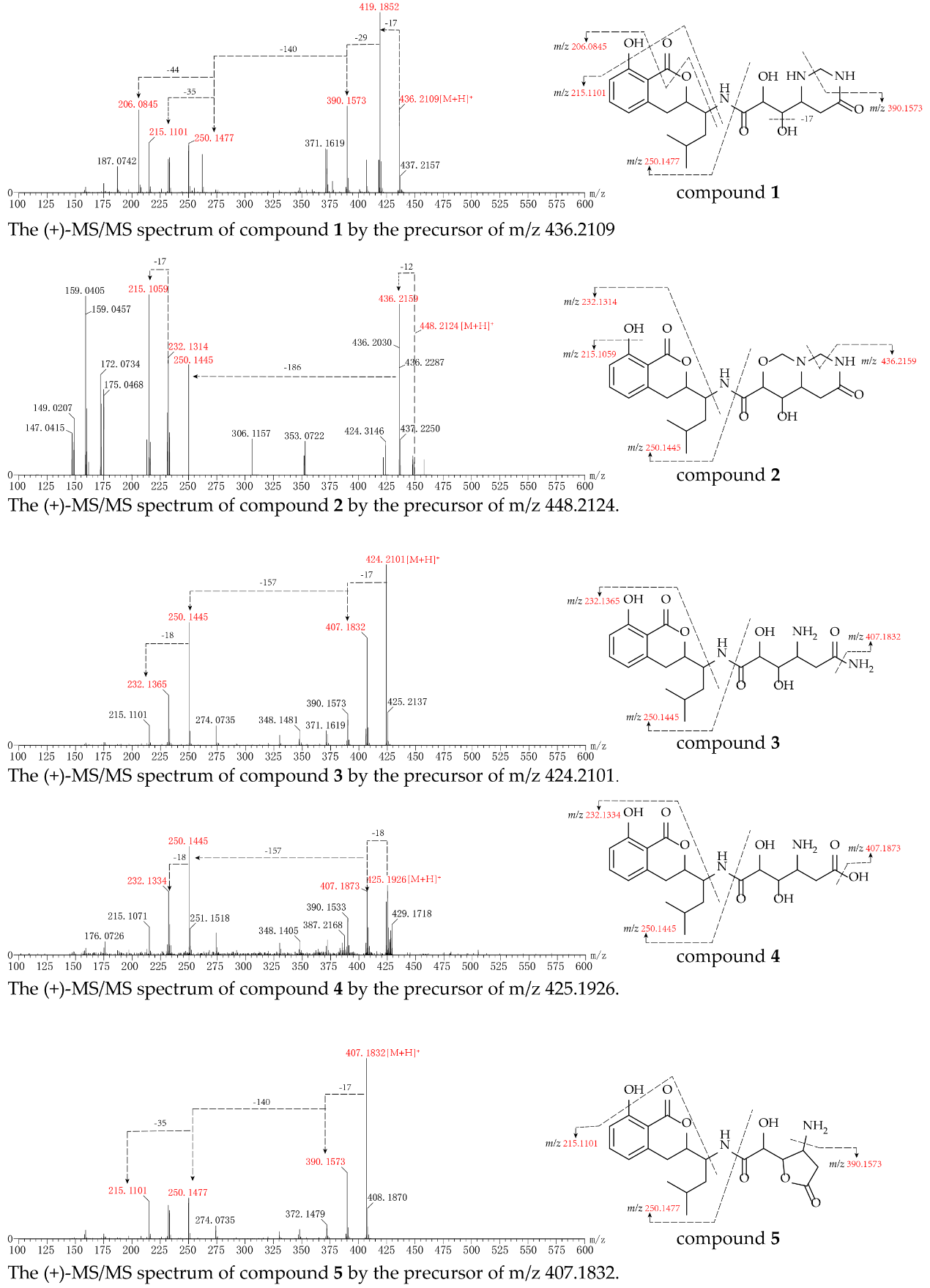


**Figure S3a.** The MS/MS spectrum and possible fragmentation patterns of compounds **1**-**5**.


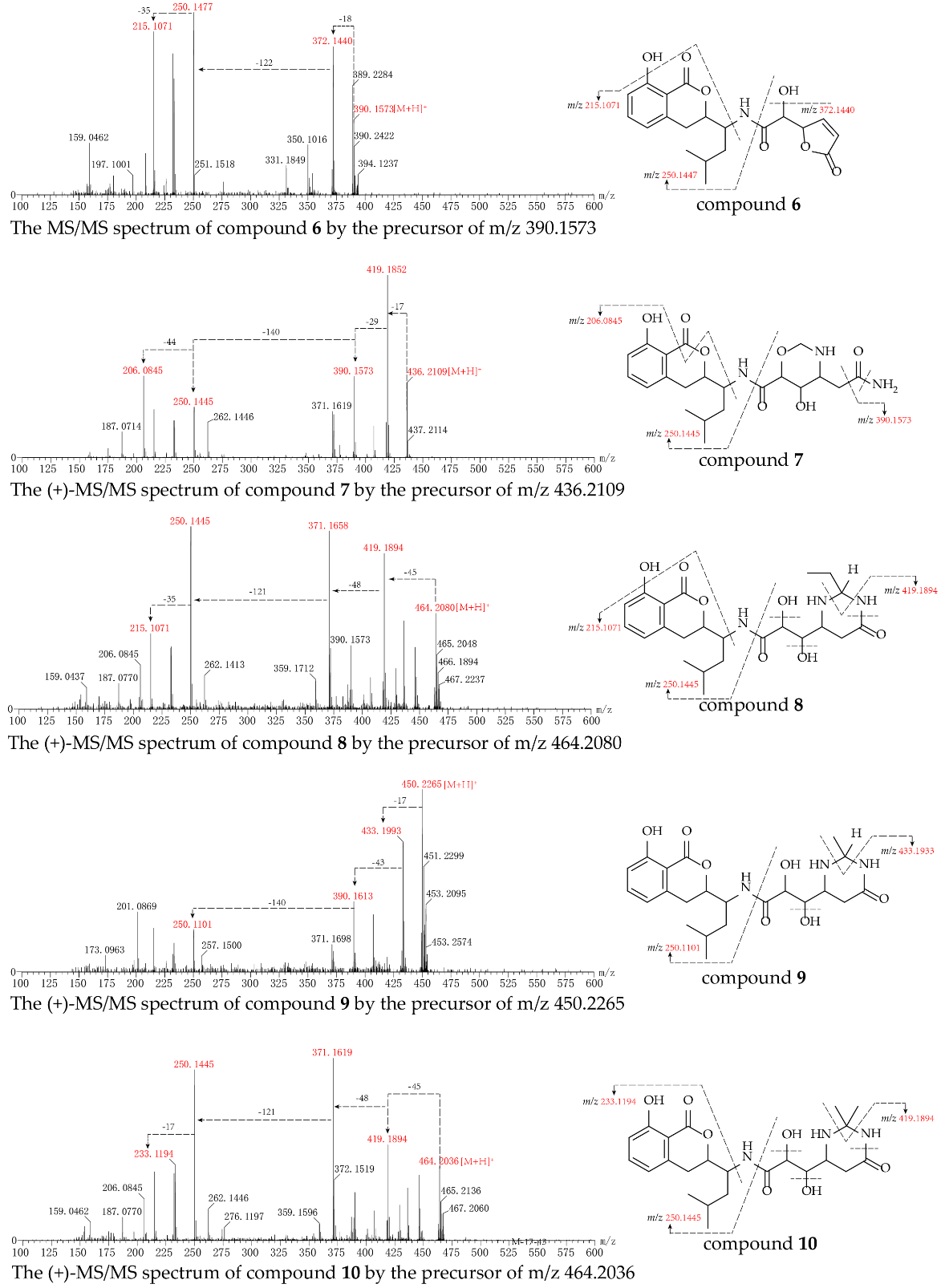


**Figure S3b.** The MS/MS spectrum and possible fragmentation patterns of compounds **6**-**10**.


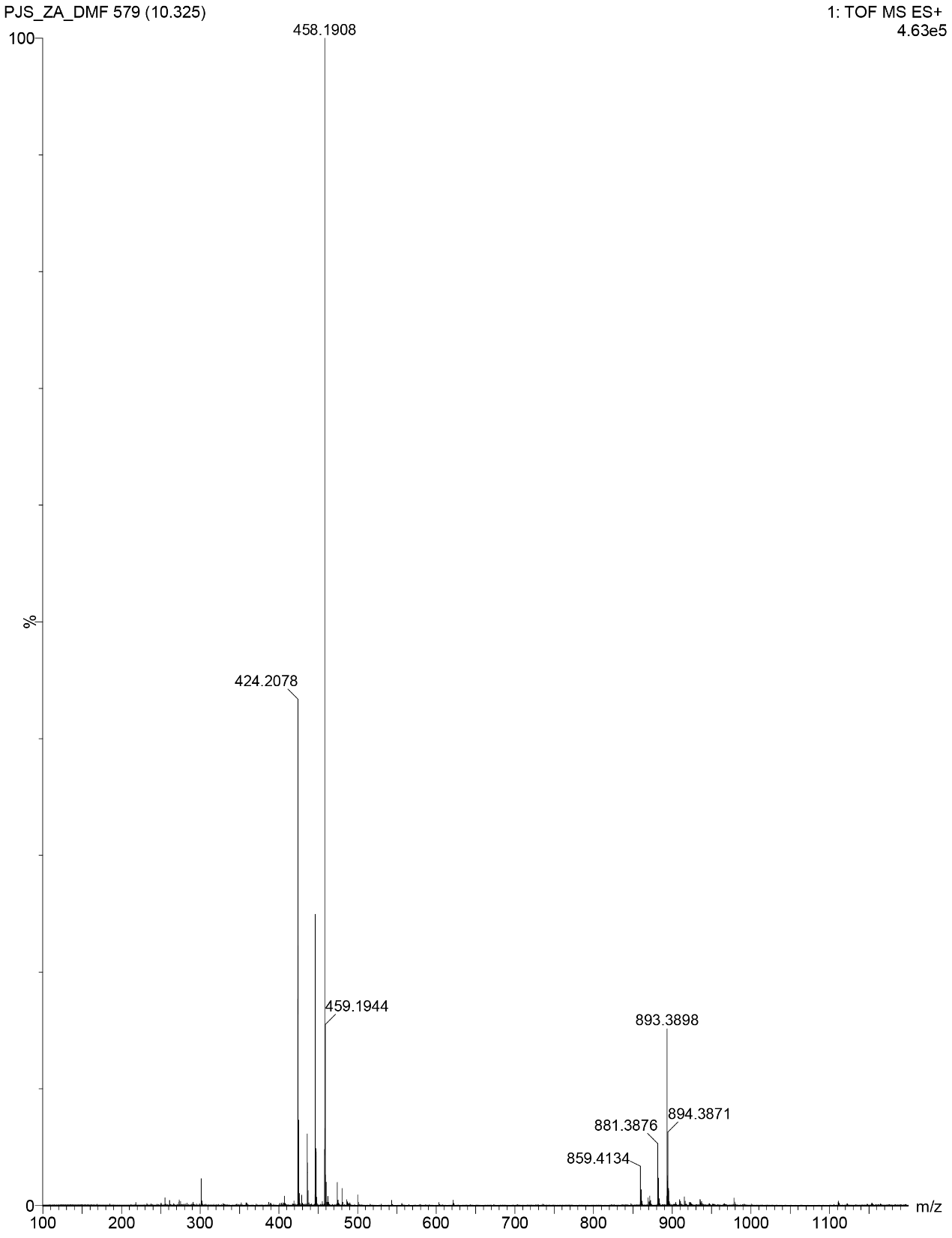


**Figure S4**. The HR-ESI-MS spectrum of compound **1.**


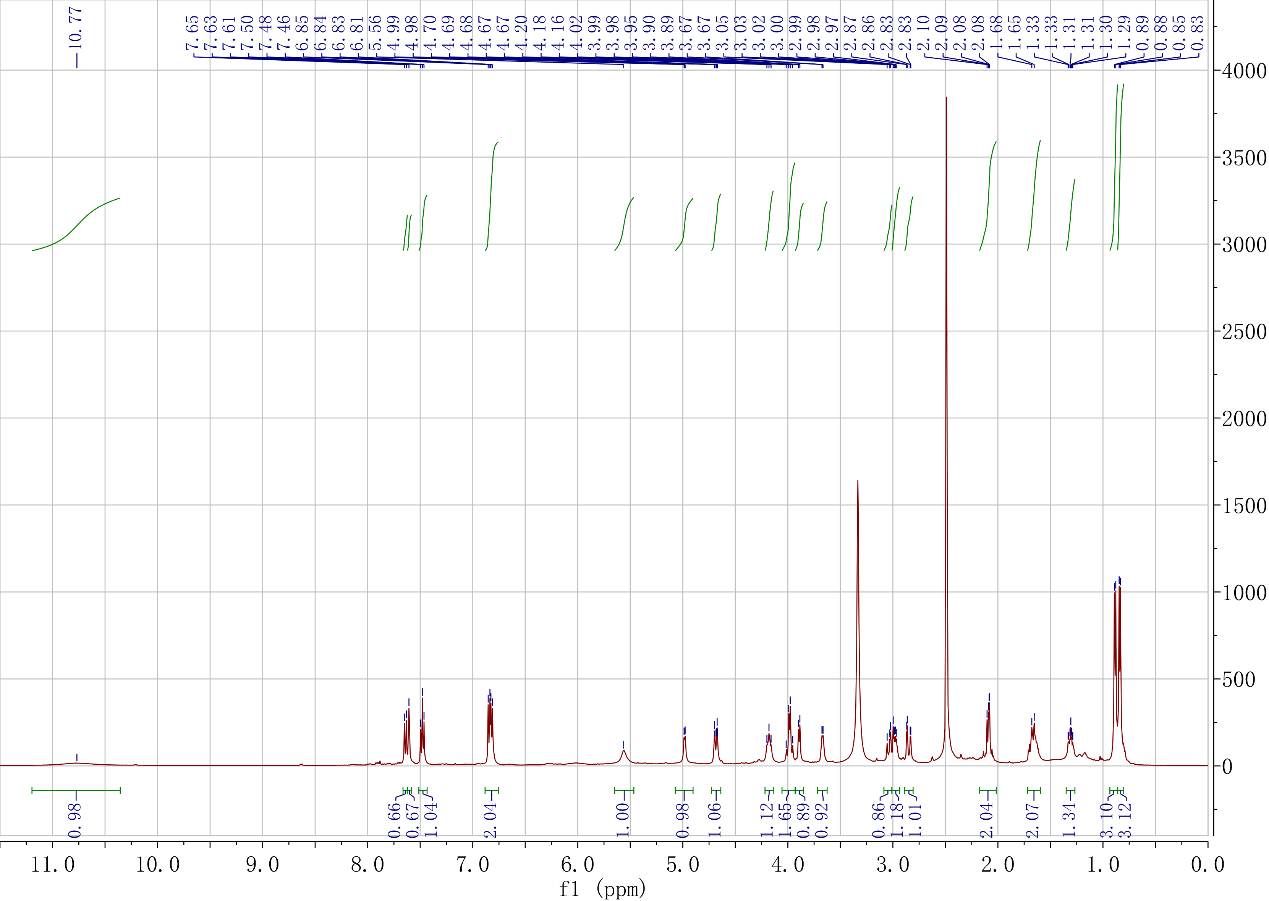


**Figure S5**. The ^1^H NMR spectrum of compound **1** in DMSO-*d_6_* (500 MHz).


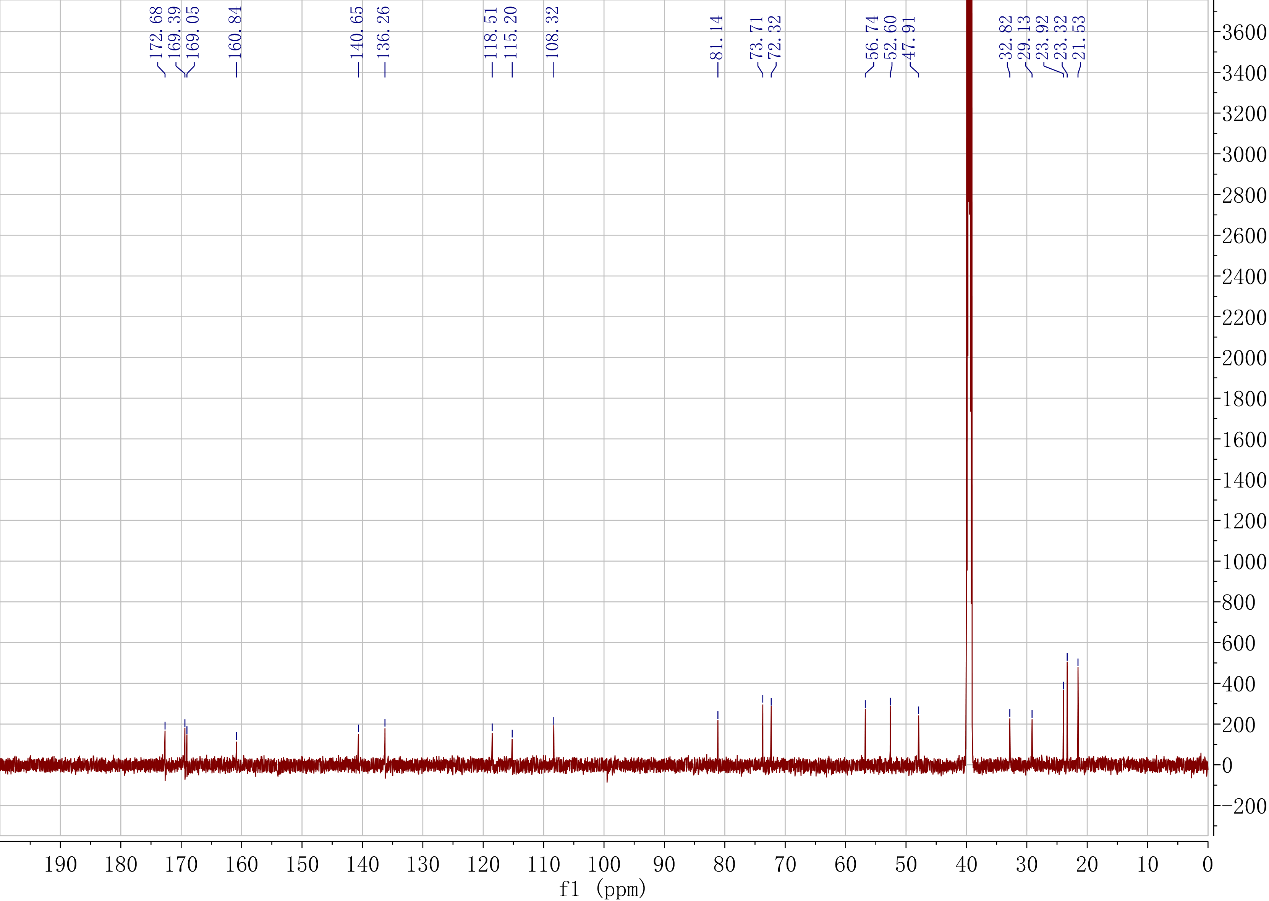


**Figure S6**. The ^13^C NMR spectrum of compound **1** in DMSO-*d_6_* (125 MHz).


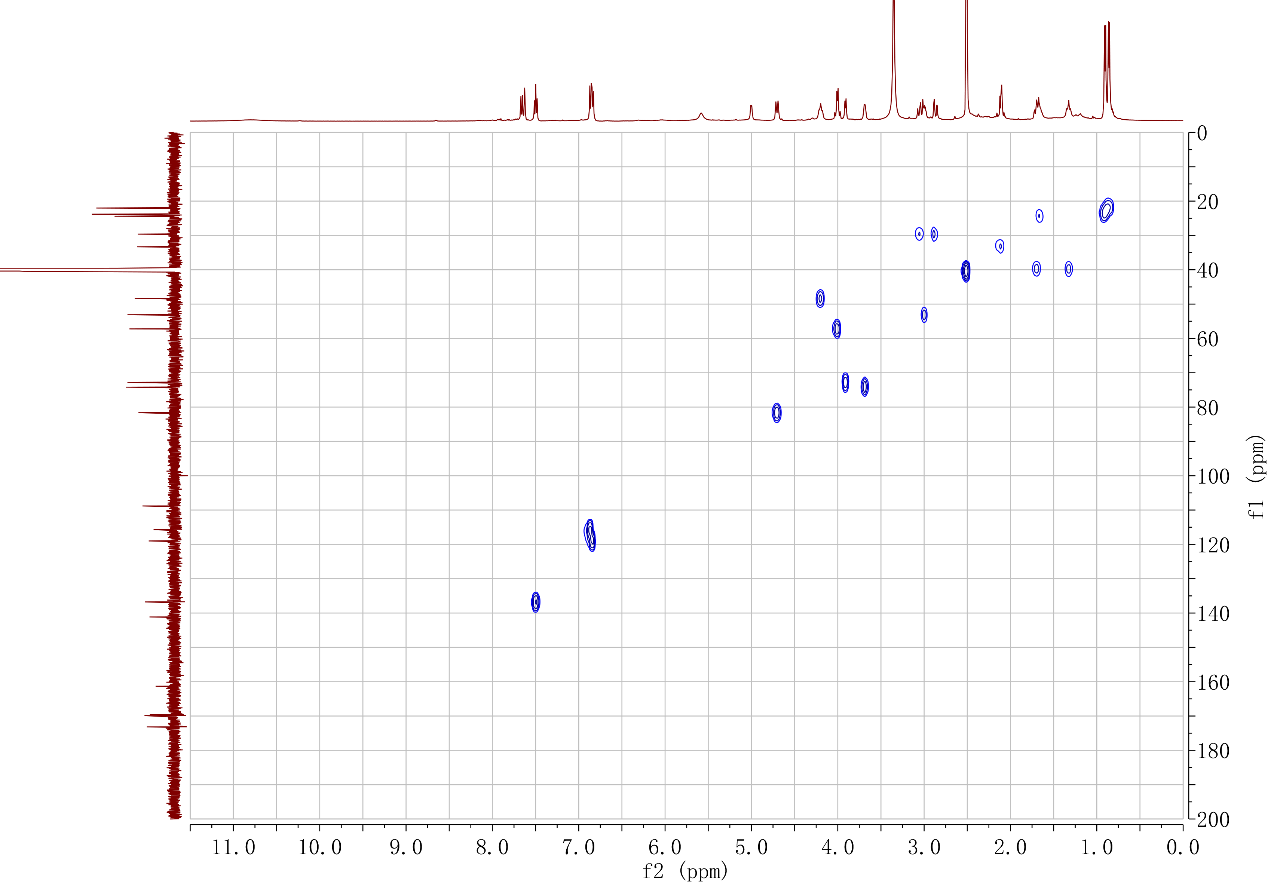


**Figure S7**. The HSQC spectrum of compound **1** in DMSO-*d_6_*.


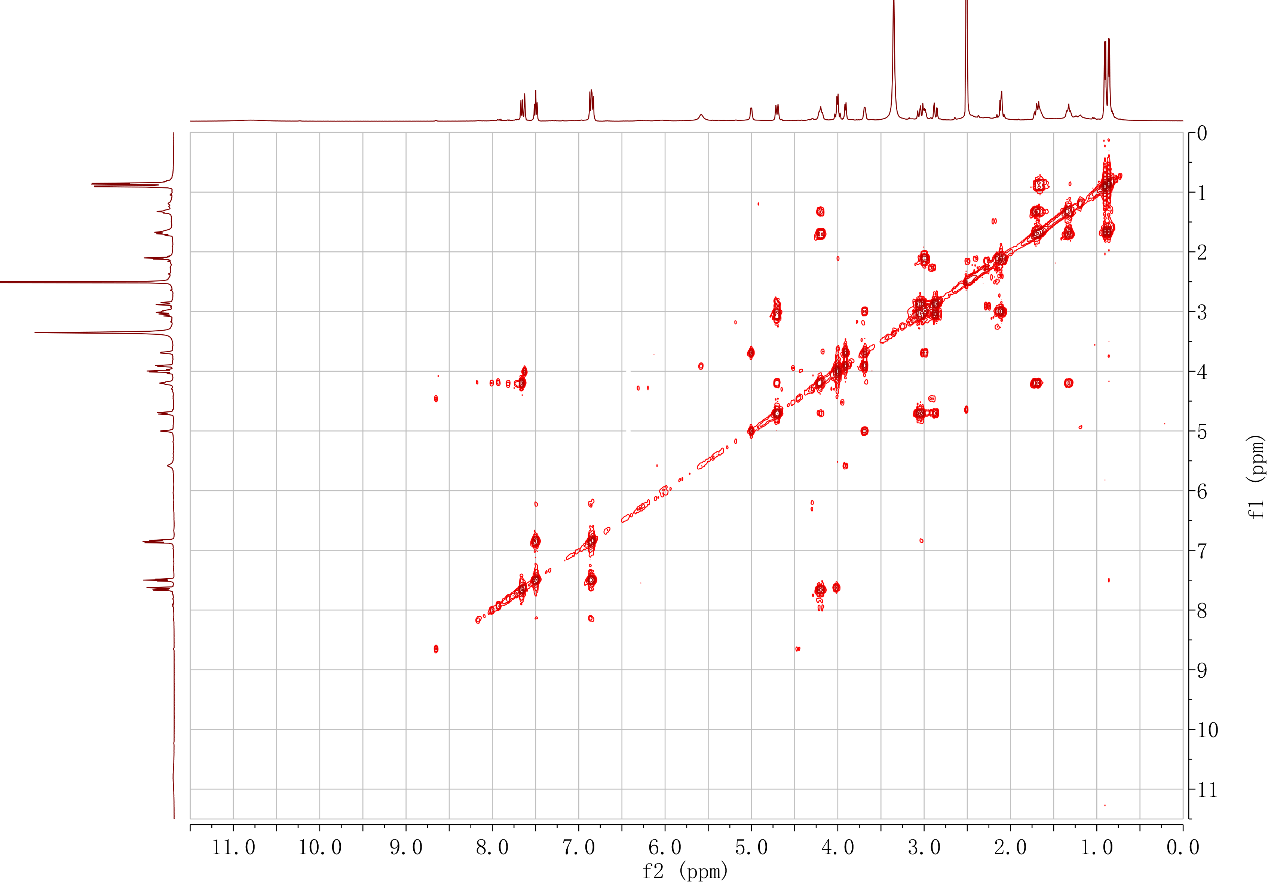


**Figure S8**. The COSY spectrum of compound **1** in DMSO-*d_6_*.


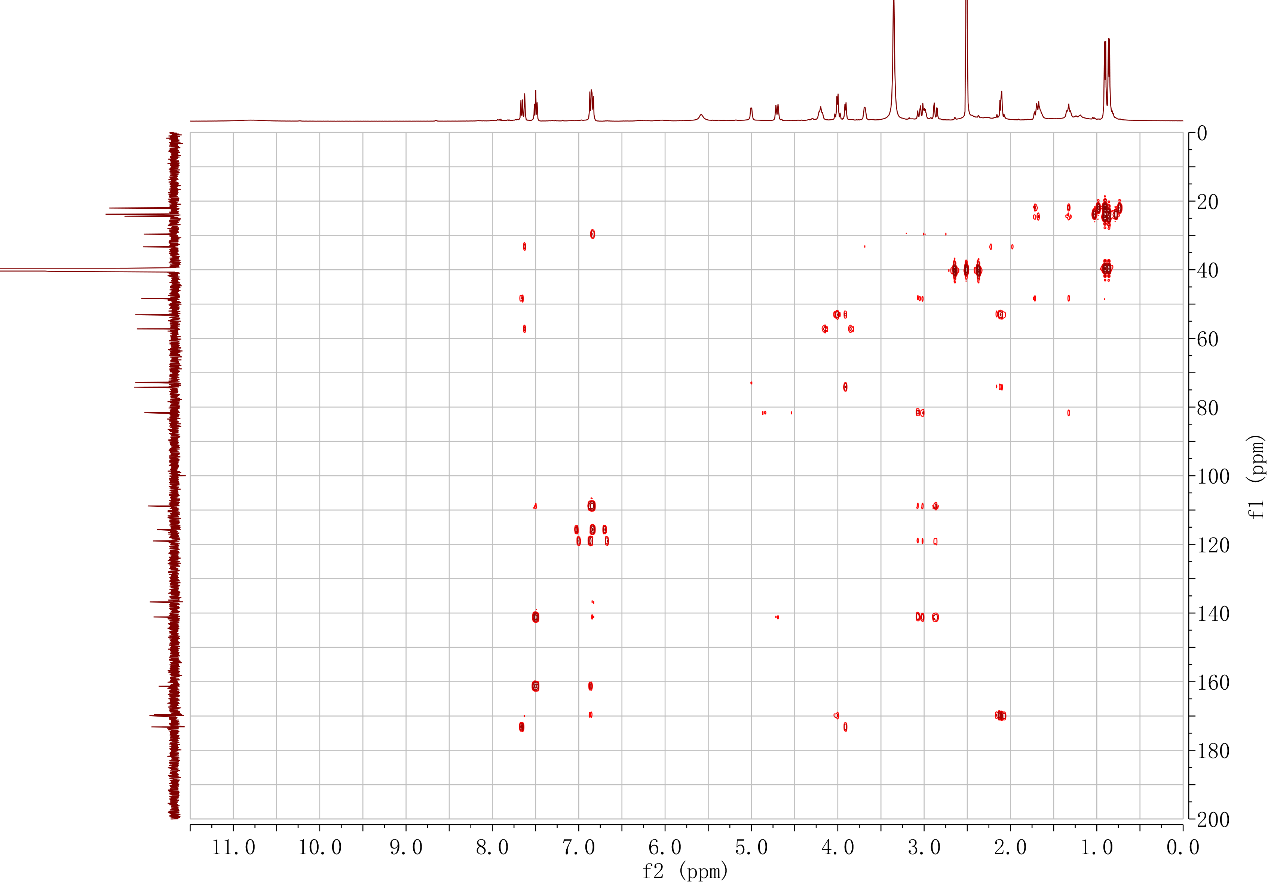


**Figure S9**. The HMBC spectrum of compound **1** in DMSO-*d_6_*.


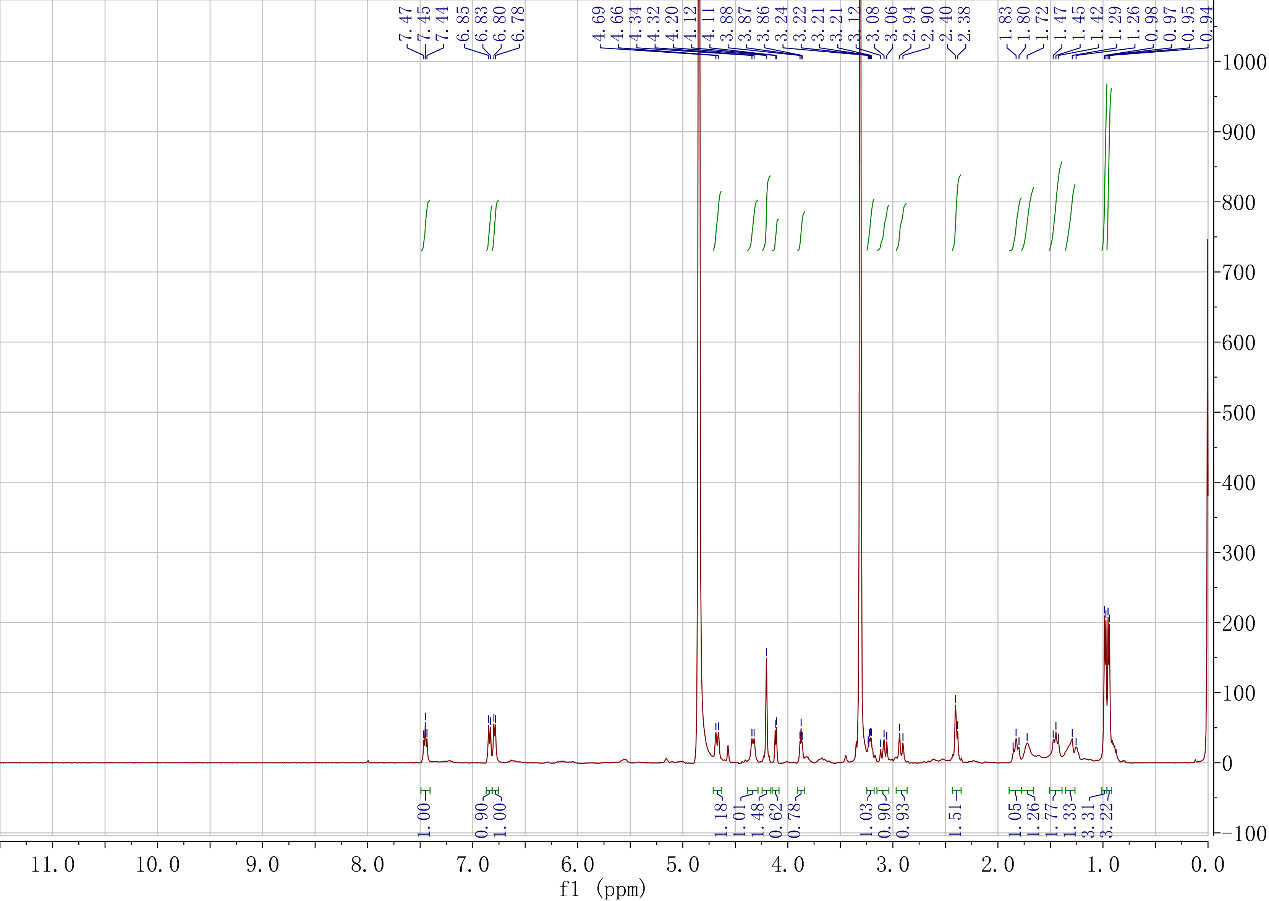


**Figure S10**. The ^1^H NMR spectrum of compound **1** in CD_3_OD (500 MHz).


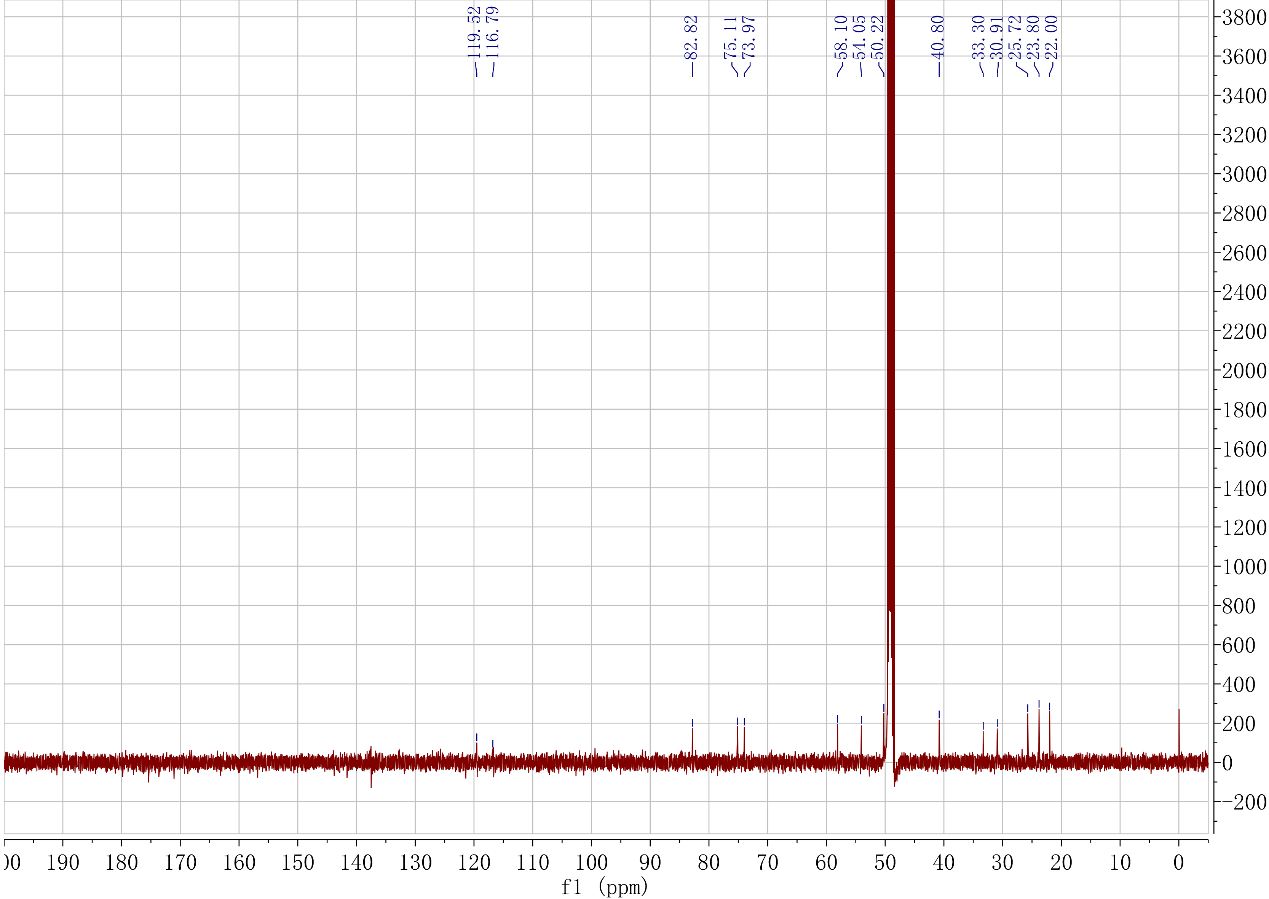


**Figure S11**. The ^13^C NMR spectrum of compound **1** in CD_3_OD (125 MHz).


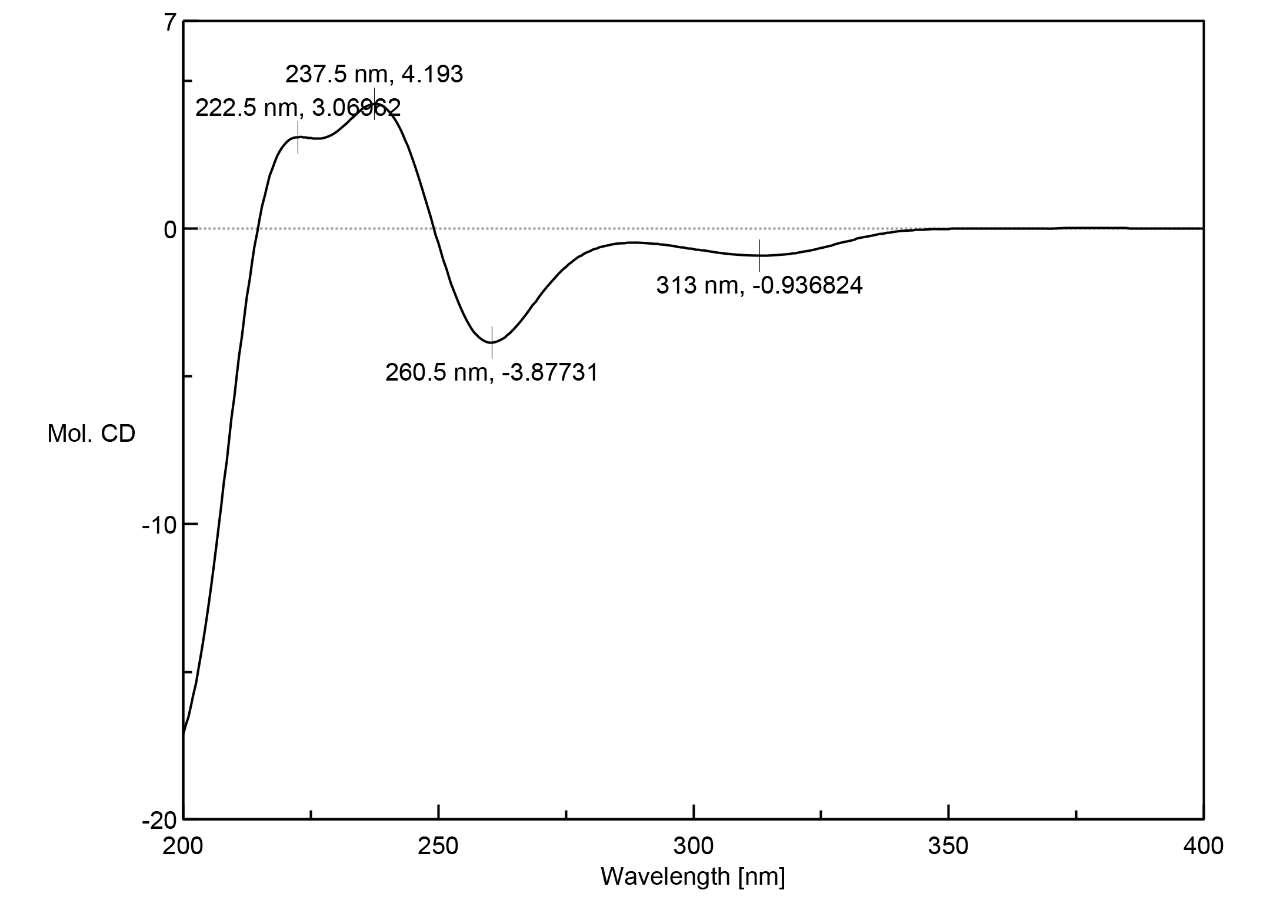


**Figure S12**. The CD spectrum of compound **1**.


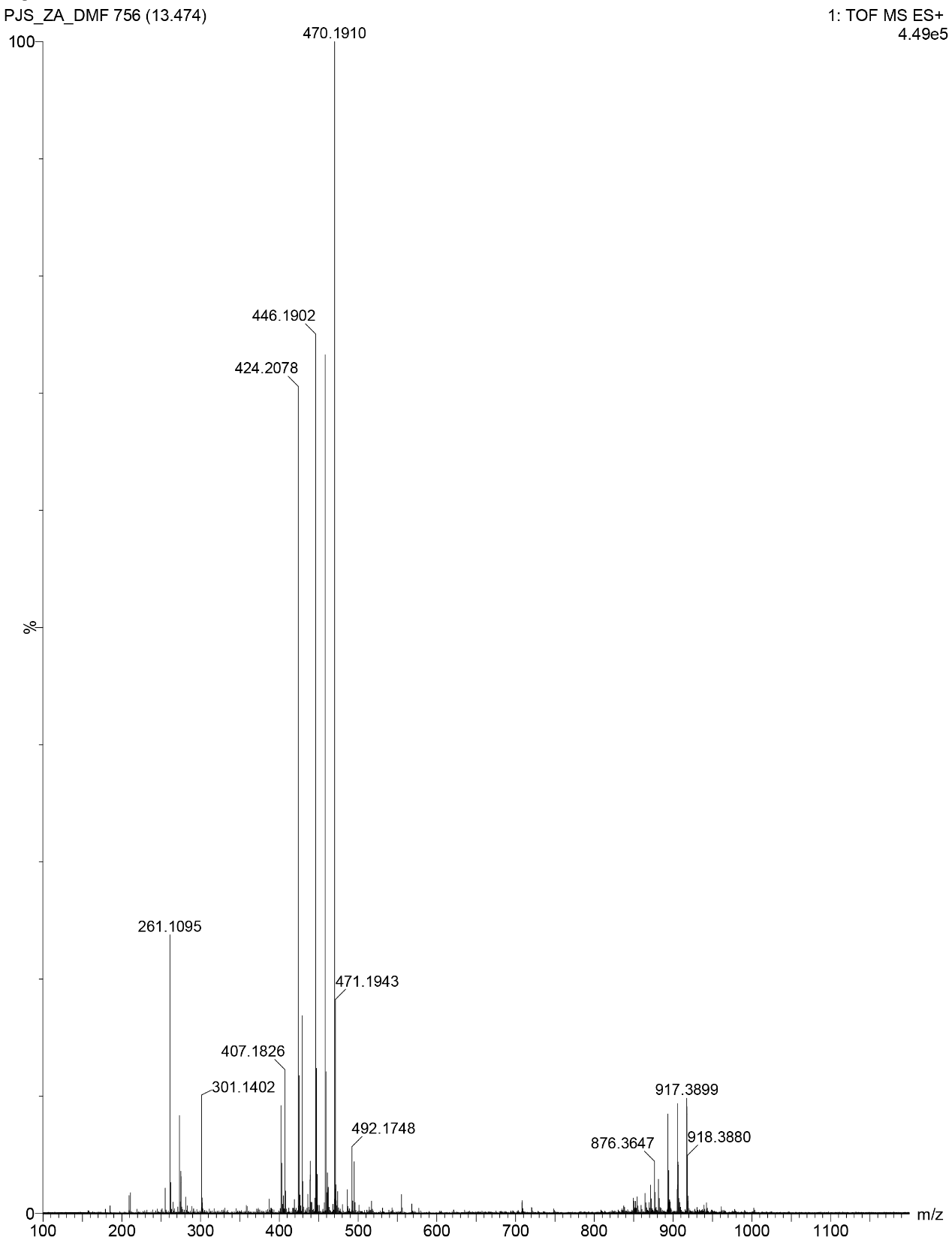


**Figure S13**. The HR-ESI-MS spectrum of compound **2**.


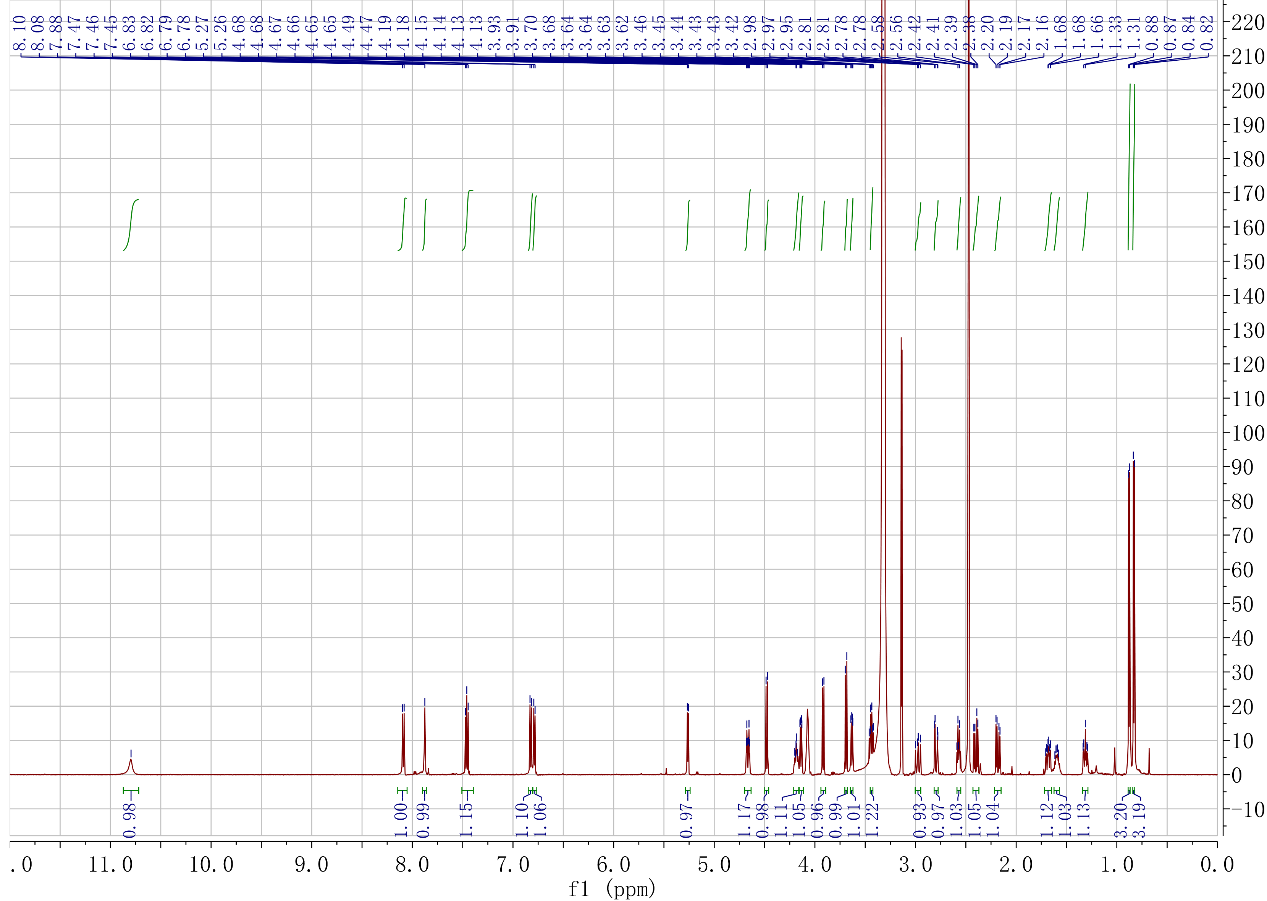


**Figure S14**. The ^1^H NMR spectrum of compound **2** in DMSO (600 MHz).


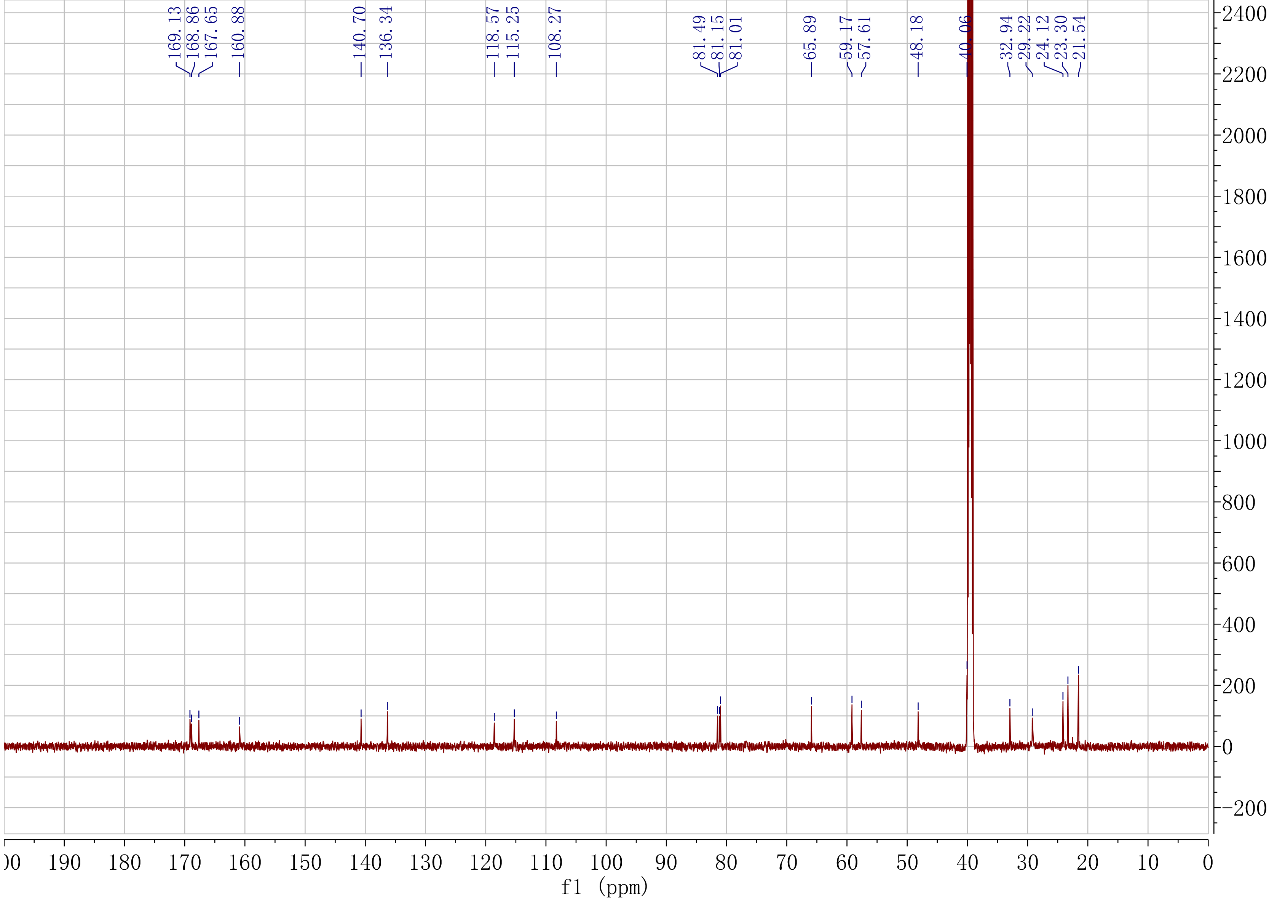


**Figure S15**. The ^13^C NMR spectrum of compound **2** in DMSO-*d_6_* (150 MHz).


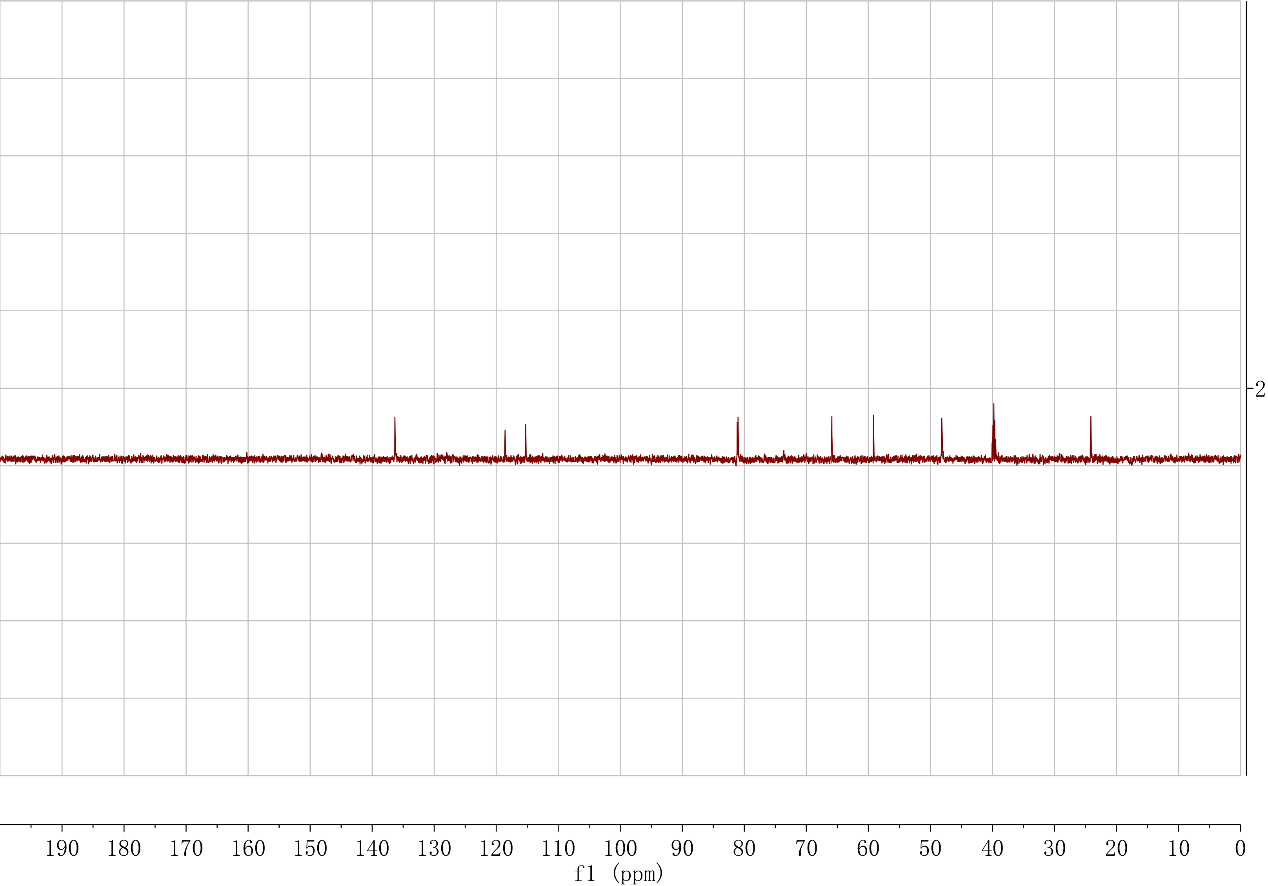


**Figure S16**. The DEPT 90 spectrum of compound **2** in DMSO-*d_6_* (150 MHz).


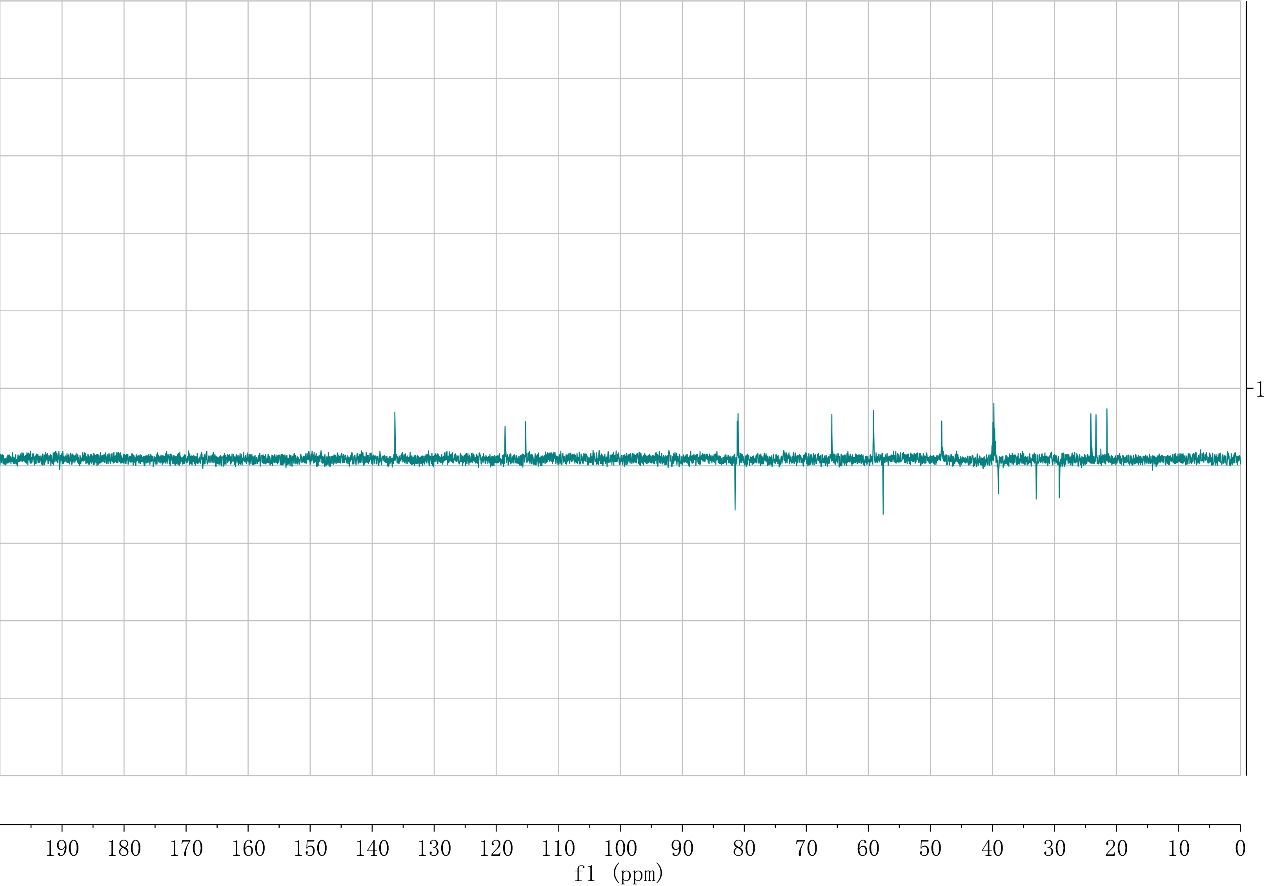


**Figure S17**. The DEPT spectrum 135 of compound **2** in DMSO-*d_6_* (150 MHz).


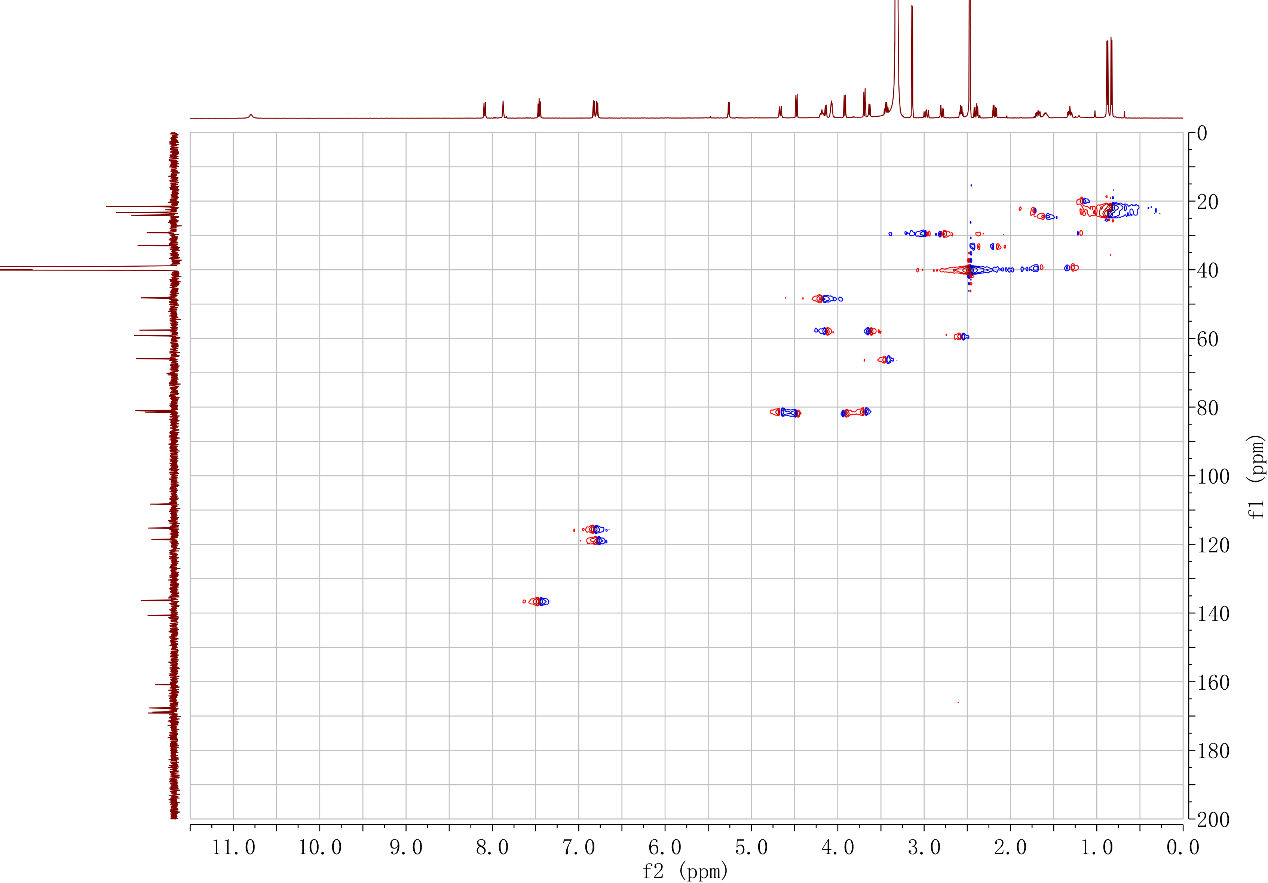


**Figure S18**. The HSQC spectrum of compound **2** in DMSO-*d_6_*.


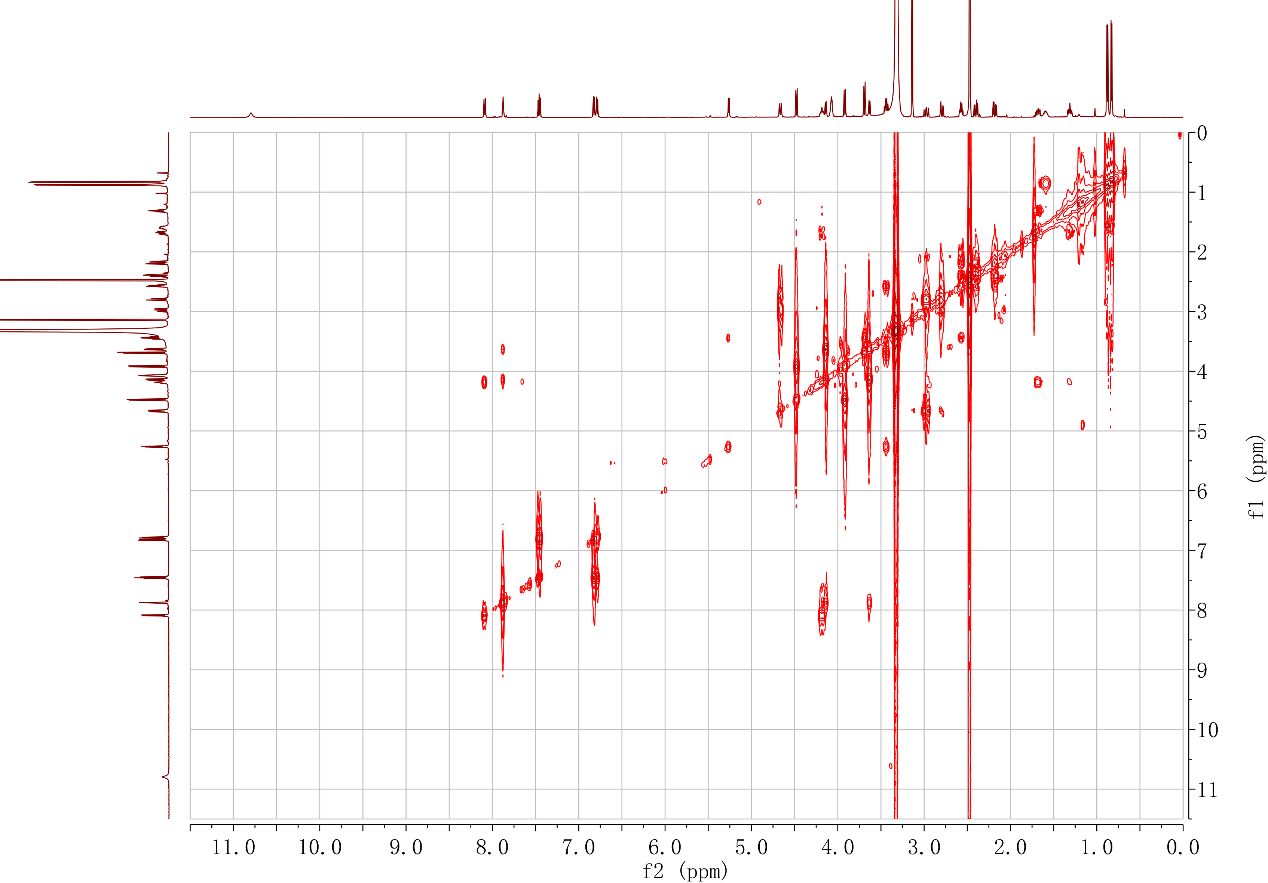


**Figure S19**. The COSY spectrum of compound **2** in DMSO-*d_6_*.


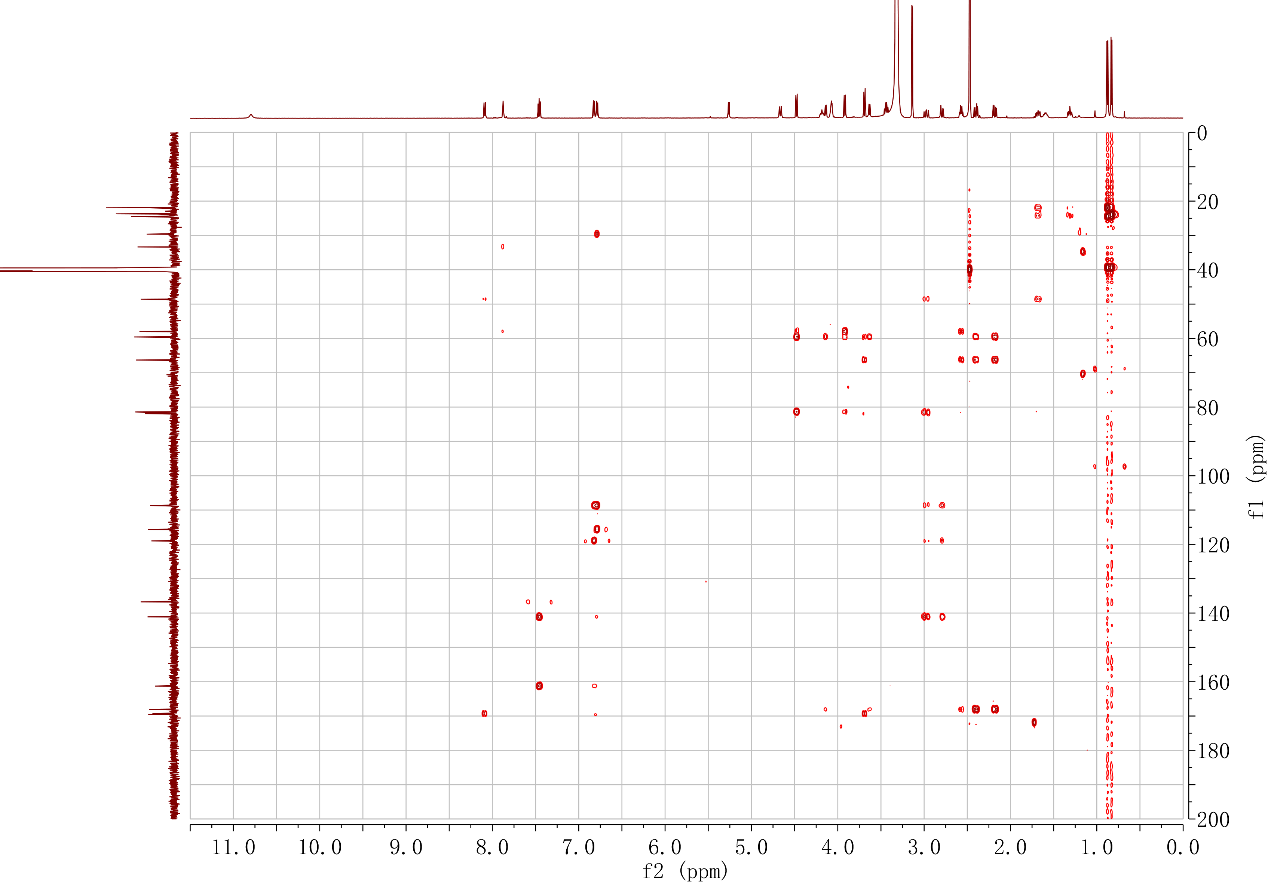


**Figure S20**. The HMBC spectrum of compound **2** in DMSO-*d_6_*.


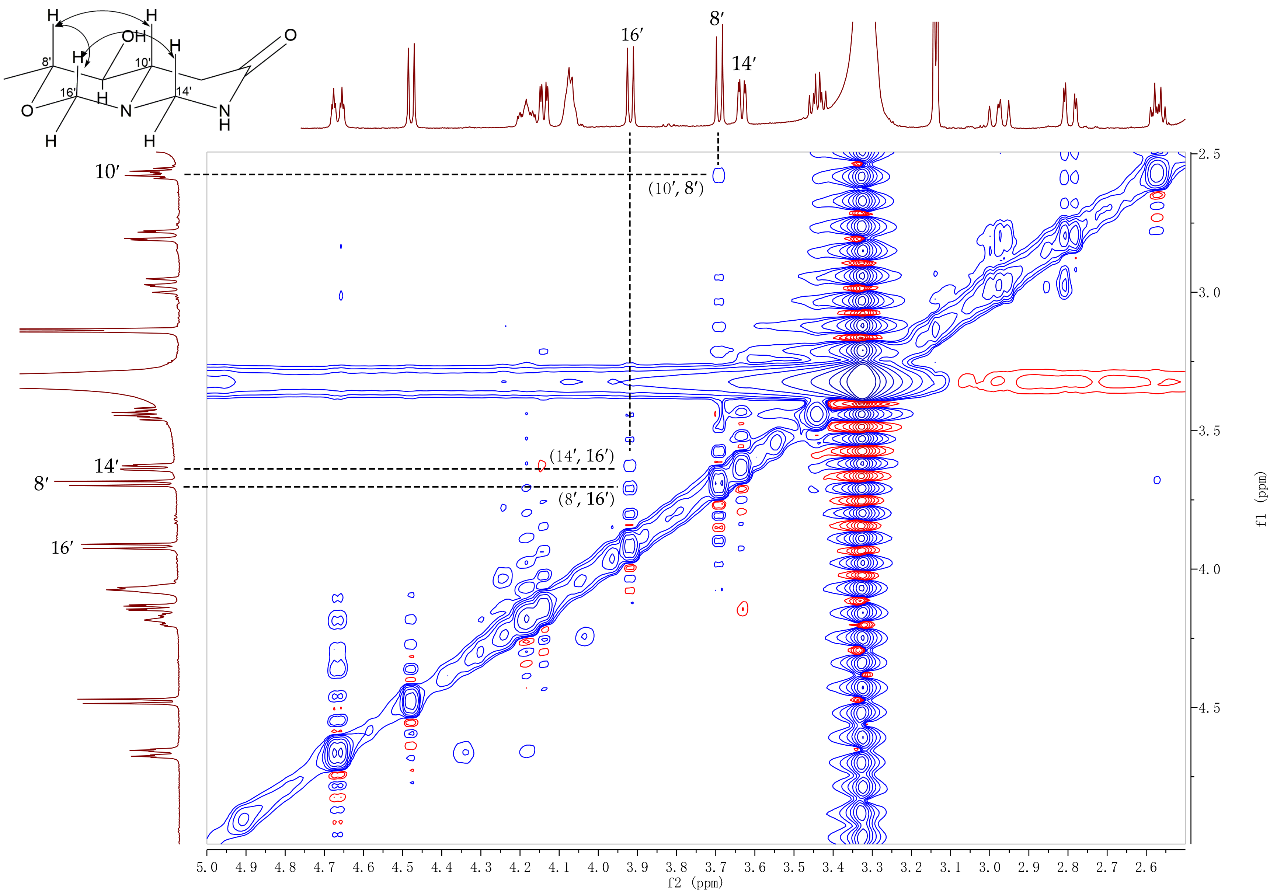


**Figure S21**. The NOESY spectrum of compound **2** in DMSO-*d_6_*.


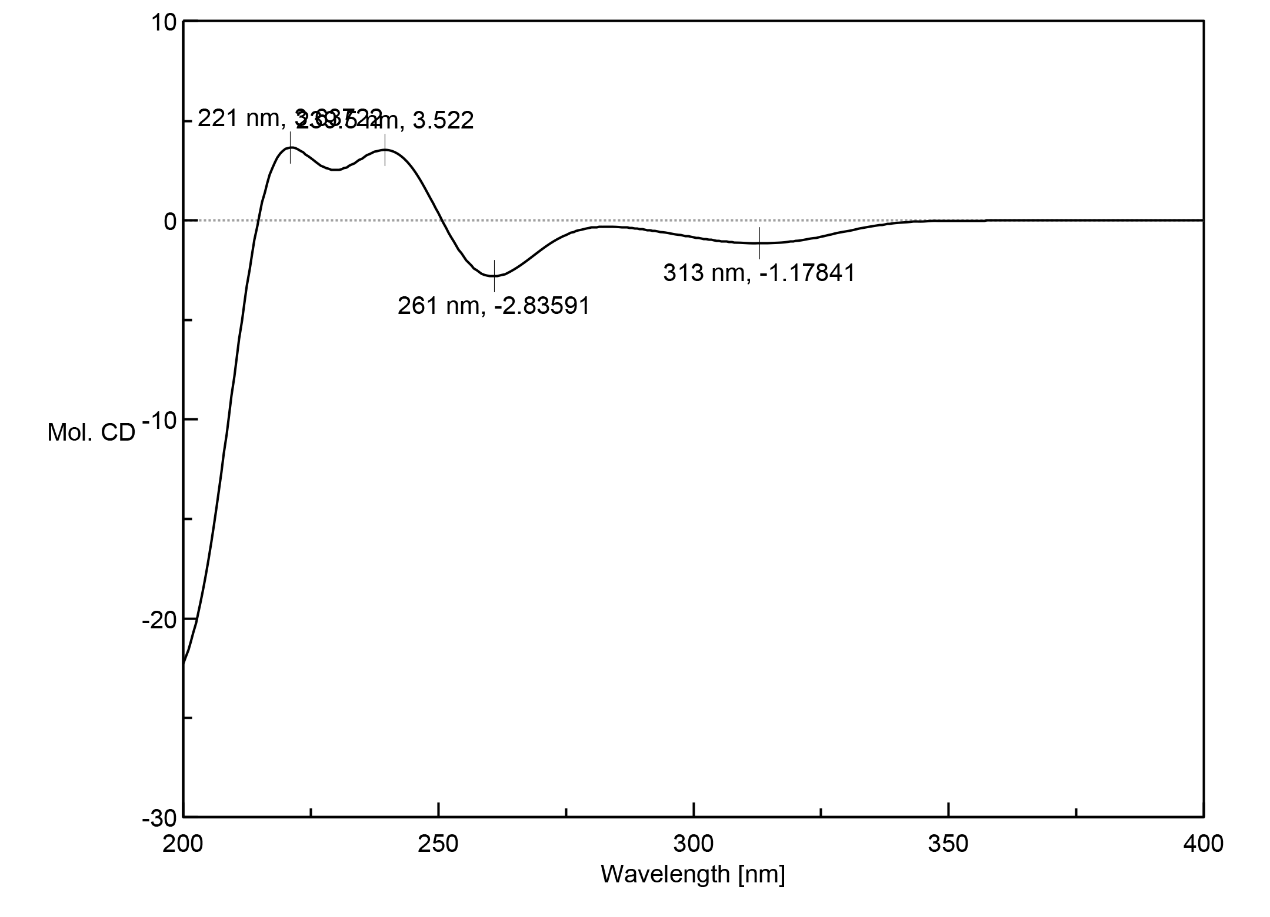


**Figure S22**. The CD spectrum of compound **2**.

**Table S1.** The MS/MS fragment ions of two new antibiotics (**1**-**2**) and compounds **3**-**10**.

| Peak No. | Observed Mass (Da) | | Calculated Mass (Da) | | Formula | | Adduct form | MS/MS Fragment Ions (*m/z*) | | | |
| --- | --- | --- | --- | --- | --- | --- | --- | --- | --- | --- | --- |
| **1** | 436.2109 | 436.2078 | | C_21_H_30_N_3_O_7_^+^ | | [M+H]^+^ | | | 206.0845 | 215.1101 | 250,1477 |
|  |  |  |  |  |  |  |  |  | 390.1573 | 419.1852 |  |
| **2** | 448.2124 | 448.2078 | | C_22_H_30_N_3_O_7_^+^ | | [M+H]^+^ | | | 215.1059 | 232.1314 | 250.1445 |
|  |  |  |  |  |  |  |  |  | 436.2159 |  |  |
| **3** | 424.2101 | 424.2078 | | C_20_H_30_N_3_O_7_^+^ | | [M+H]^+^ | | | 232.1365 | 250.1445 | 407.1832 |
| **4** | 425.1926 | 425.1918 | | C_20_H_29_N_2_O_8_^+^ | | [M+H]^+^ | | | 232.1334 | 250.1445 | 407.1873 |
| **5** | 407.1832 | 407.1813 | | C_20_H_27_N_2_O_7_^+^ | | [M+H]^+^ | | | 215.1101 | 250.1447 | 390.1573 |
| **6** | 390.1573 | 390.1553 | | C_20_H_24_NO_7_^+^ | | [M+H]^+^ | | | 215.1071 | 250.1477 | 372.1440 |
| **7** | 436.2109 | 436.2078 | | C_21_H_30_N_3_O_7_^+^ | | [M+H]^+^ | | | 206.0845 | 250.1445 | 390.1573 |
|  |  |  |  |  |  |  |  |  | 419.1852 |  |  |
| **8** | 464.2080 | 464.2391 | | C_23_H_34_N_3_O_7_^+^ | | [M+H]^+^ | | | 215.1071 | 250.1445 | 371.1658 |
|  |  |  |  |  |  |  |  |  | 419.1894 |  |  |
| **9** | 450.2265 | 450.2234 | | C_22_H_32_N_3_O_7_^+^ | | [M+H]^+^ | | | 250.1101 | 390.1613 | 433.1993 |
| **10** | 464.2036 | 464.2391 | | C_23_H_34_N_3_O_7_^+^ | | [M+H]^+^ | | | 233.1194 | 250.1445 | 371.1619 |
|  |  |  |  |  |  |  |  |  | 419.1894 |  |  |

**Table S2.** Minimum inhibitory concentrations (MICs) of hetiamacin E (**1**) and hetiamacin F (**2**)

| Test organisms | MICs (µg/mL) | | |
| --- | --- | --- | --- |
|  | hetiamacin E (**1**) | hetiamacin F (**2**) | levofloxacin |
| *S. epidermidis* ATCC 12228 (MSSE) | 2 | 32 | 0.25 |
| *S. epidermidis* 16–4 (MSSE) | 4 | 32 | 0.25 |
| *S. epidermidis* 16–5 (MSSE) | 4 | 32 | 8 |
| *S. aureus* ATCC 29213 (MSSA) | 8 | >32 | 0.25 |
| *S. aureus* ATCC 33591 (MRSA) | 16 | >32 | 0.25 |
| *S. aureus* 15 (MSSA) | >64 | >32 | 0.25 |
| *S. aureus* 16-1 (MSSA) | >64 | >32 | 0.25 |
| *S. aureus* 16-30 (MRSA) | >64 | >32 | 16 |
| *Enterococcus faecalis* ATCC 29212 (VSE) | >64 | >32 | 1 |
| *E. faecalis* ATCC 51299 (VRE) | >64 | >32 | 1 |
| *E. faecalis* ATCC 51575 (VRE) | >64 | >32 | 1 |
| *E. faecalis* 16-6 (VSE) | >64 | >32 | 2 |
| *E. faecium* ATCC 700221 (VRE) | 64 | >32 | 32 |
| *E. faecium* 16-5 (VSE) | >64 | >32 | 32 |
| *E. faecium* 12-1 (VRE) | >64 | >32 | 64 |
| *Escherichia coli* ATCC 25922 (ESBLs (-)) | 64 | >32 | ≤0.03 |
| *Escherichia coli* ATCC 35218 (ESBLs (+)) | >64 | >32 | ≤0.03 |
| *Escherichia coli* ATCC 2469 (NDM-1(+)) | >64 | >32 | >128 |
| *Escherichia coli* 16-1 (ESBLs (+)) | >64 | >32 | 0.5 |
| *Escherichia coli* 16-7 (ESBLs (-)) | >64 | >32 | 0.5 |
| *Klebsiella pneumonia* ATCC 700603 (ESBLs (+)) | >64 | >32 | 1 |
| *Klebsiella pneumoniae* ATCC BAA–2146 (NDM-I (+)) | >64 | >32 | >128 |
| *Klebsiella pneumonia* 16-2 (ESBLs (-)) | >64 | >32 | 0.06 |
| *Klebsiella pneumonia* 16-14 (ESBLs (+)) | >64 | >32 | 0.5 |
| *Pseudomonas aeruginosa* ATCC 27853 | >64 | >32 | 2 |
| *Pseudomonas aeruginosa* PAO1 | 64 | 32 | 4 |
| *Pseudomonas aeruginosa* 16–11 | >64 | >32 | 1 |
| *Acinetobacter baumannii* ATCC 19606 | 64 | 32 | 0.25 |
| *Enterobacter cloacae* ATCC 43560 | >64 | >32 | ≤0.03 |
| *Enterobacter aerogenes* ATCC 13048 | >64 | >32 | 0.06 |
| *Serratia marcescens*ATCC 21074 | >64 | >32 | 0.25 |
| *Citrobacter freundii* ATCC 43864 | >64 | >32 | 0.06 |
| *Providentia rettgeri* ATCC 31052 | >64 | >32 | ≤0.03 |
| *Proteus vulgaris* ATCC 29905 | >64 | >32 | ≤0.03 |
| *Proteus mirabilis* ATCC 49565 | >64 | >32 | 0.06 |
| *Stenotrophomonas maltophilia* ATCC 13636 | >64 | >32 | 2 |
| *Shigella flexneri* ATCC 12022 | 64 | >32 | ≤0.03 |

Note: MSSE, methicillin–susceptible *Staphylococcus epidermidis*; MRSE, methicillin–resistant *Staphylococcus epidermidis*; MSSA, methicillin–susceptible *Staphylococcus aureus*; MRSA, methicillin–resistant *Staphylococcus aureus*; VSE, vancomycin sensitive *Enterococcus*; VRE, vancomycin–resistant *Enterococcus*; ESBLs, extended-spectrum β-lactamases; NDM-I, New Delhi metallo-β-lactamase I.
